# Triple Matrix Confinement‐Induced Ultrabright Afterglow From Carbon Dots With Multivariate Responsive Afterglow Colors for Advanced Dynamic Information Encryption

**DOI:** 10.1002/anie.8266335

**Published:** 2026-03-02

**Authors:** Yupeng Liu, Yiming Hao, Yi Li, Xiaofan Xia, Xichang Ou, Xue Wu, Jun Wu, Ruifeng Zheng, Dongbo Guo, Shi Chen, Jing Li, Jinyang Zhu, Qijun Li, Songnan Qu

**Affiliations:** ^1^ Institute of Applied Physics and Materials Engineering (IAPME) Joint Key Laboratory of Ministry of Education University of Macau Taipa Macau SAR China; ^2^ Zhuhai UM Science and Technology Research Institute University of Macau Taipa Macau SAR China; ^3^ Department of Physics and Chemistry Faculty of Science and Technology University of Macau Taipa Macau SAR China; ^4^ School of Mechanical Engineering Jiangsu University Zhenjiang China; ^5^ State Centre for International Cooperation on Designer Low‐Carbon & Environmental Materials School of Materials Science and Engineering Zhengzhou University Zhengzhou China; ^6^ School of Chemical Engineering and Light Industry Guangdong University of Technology Guangzhou China; ^7^ State Key Laboratory of Digital Medical Engineering School of Biomedical Engineering Sanya Research Institute of Hainan University Hainan University Sanya China; ^8^ School of Mechanical Engineering Institute of Technology for Carbon Neutralization Yangzhou University Yangzhou China

**Keywords:** carbon dots, excitation‐dependent afterglow colors, thermochromic afterglow, time‐dependent afterglow colors, ultra‐bright afterglow

## Abstract

Dynamic afterglow carbon dots (CDs) materials, capable of long‐duration emission and dynamic color changes after excitation, hold promise for widespread applications in high‐level encryption and visual sensing. However, the single‐variable response afterglow color changing limits the potential of CDs for multidimensional information encoding and dynamic encryption in complex environments. Here, we report metal‐free CDs with triple‐variable responses (time, temperature, and excitation wavelength) for multiple dynamic afterglow colors and an ultra‐high afterglow brightness (406 cd m^−2^) far exceeding those of other afterglow materials. Based on the synergistic effect of triple matrix confinement and interface effects, room‑temperature phosphorescence (RTP) and thermally activated delayed fluorescence (TADF) are integrated into a single CD system. Owing to the different lifetimes of the dual‐mode afterglow (phosphorescence and TADF) and their varying sensitivities to temperature and excitation wavelength, time‐dependent afterglow colors (TDAC), excitation‐dependent afterglow colors (EDAC), and thermochromic afterglow (TCAG) are simultaneously achieved. Finally, we also designed a three‐dimensional variable‐response color code that varies with time, temperature, and excitation wavelength, spanning the entire visible spectrum. This platform enables high‐capacity, programmable, full‐gamut, and visually intuitive information encryption and display, providing a promising pathway for advanced, multidimensional anti‐counterfeiting and secure communication technologies.

## Graphical abstract

A novel carbon dots material (BUL‐CDs) with an initial afterglow brightness (406 cd m^−2^) far exceeding that of other afterglow luminescent materials was developed. Furthermore, the BUL‐CDs exhibited time‐dependent afterglow colors, excitation‐dependent afterglow colors, and thermochromic afterglow in response to triplet variables. BUL‐CDs‐inked 3D color codes were designed to achieve high‐order, multi‐dimensional information encryption.

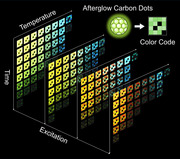

## 1 Introduction

Afterglow materials (also referred to as persistent luminescent materials), which continue to emit light after the cessation of external excitation, have emerged as versatile platforms for applications in information storage and retrieval, optical anti‐counterfeiting, visual sensing, flexible optoelectronics, and bioimaging [1, 2]. Based on their luminescence mechanisms, afterglow materials are generally categorized into long‐persistent luminescence (LPL), room‐temperature phosphorescence (RTP), and thermally activated delayed fluorescence (TADF) [3]. Responsive dynamic afterglow materials integrate persistent luminescence with controllable color modulation, enabling prolonged light emission after excitation and dynamic color variation in response to external variables [4]. These unique properties offer significant advantages for low‐energy visualization, passive nighttime displays, bioimaging, anti‐counterfeiting, and information encryption. On one hand, the intrinsic persistence effect of afterglow ensures information readability without continuous excitation, reducing energy consumption and enhancing concealment [5]. On the other hand, tunable color and spatiotemporal programmability allow multidimensional information encoding, thereby improving security and anti‐counterfeiting performance. By manipulating processes such as trap‐level engineering, intersystem crossing (ISC)/reverse ISC (RISC), and spin–orbit coupling (SOC), it is possible to balance lifetime, brightness, and color to some extent [6]. Nevertheless, achieving a wide color gamut, programmable, and retrievable color‐changing afterglow remains a formidable challenge [7]. Consequently, the development of color‐changing afterglow materials with high brightness, controllable color, and multi‐stimuli responsiveness holds substantial theoretical and practical significance for advancing optoelectronic display and information security technologies.

Current afterglow materials research spans rare‐earth‐doped inorganic phosphors [8], organic‐inorganic hybrids [9], and organic small‐molecule or polymer systems [10]. Inorganic long‐afterglow materials (e.g., doped metal crystals and halide perovskites) generally offer long lifetimes and high efficiency but suffer from reliance on heavy/rare metal elements, limited color‐tuning flexibility, and costly processing. Organic RTP and polymeric systems provide facile processing and structural tunability but are vulnerable to oxygen quenching and thermally activated non‐radiative decay, typically requiring rigid or confined matrices to preserve emission [11]. These intrinsic limitations hinder scalable, multimodal afterglow encoding. Against this background, carbon dots (CDs) have emerged as attractive carbon‐based luminophores owing to mild synthesis conditions, low cost, favorable biocompatibility, and widely tunable surface chemistry [12]. CDs also afford rich opportunities for engineering surface trap states and for tuning fluorescence/phosphorescence pathways, offering a promising route to programmable afterglow [13]. Nevertheless, despite these advantages, most reported CD‐based afterglow systems remain static and single‐color, with research largely focused on enhancing brightness or extending lifetime. Realizing controllable dynamic afterglow colors in CDs is still in its infancy.

Recent advances have demonstrated progress in both fundamental mechanisms and practical applications of afterglow CDs [14]. Strategies such as matrix confinement/covalent anchoring [15, 16], heavy‐atom (halogen) coupling [17], and host–guest [10] or defect/surface‐state engineering [18] have enabled phosphorescence or TADF at room temperature, responsive to variables such as time or temperature [19]. Nevertheless, most reported afterglow CDs exhibit only single‐mode responsiveness—for instance, time‐dependent color evolution [20, 21, 22, 23], or thermochromic behavior [19, 24, 25]. For example, our group first reported time‐dependent phosphorescence colors transition from orange to green in CDs in 2021 [20]. Subsequently, in 2024, we achieved yellow RTP and blue TADF within a single CDs system, demonstrating the first thermochromic afterglow (TCAG) CDs‐inked paper that displayed yellow phosphorescence at low temperature and blue delayed fluorescence at high temperature [24]. Recently, Lu et al. achieved a customized dynamic time‐dependent afterglow covering the entire visible light region by adjusting the TADF and phosphorescence of CDs [26]. However, such “single‐response” systems restrict the potential of CDs for multidimensional information encoding and dynamic encryption in complex environments. To achieve multi‐responsive dynamic afterglow colors, it is essential to simultaneously regulate energy‐level structures, trap‐state distributions [22], and confinement processes at the nanoscale [23], enabling independent responses to different stimuli while collaboratively producing distinguishable color outputs. Therefore, developing a single CD system that combines ultra‐high afterglow brightness with programmable color changes under multi‐responsive variables remains a critical challenge and an unresolved research gap.

Herein, ultrabright CDs with an initial afterglow brightness of 406 cd·m^−2^ were reported, substantially higher than previously reported luminescent afterglow materials like CDs‐based, organic‐based, and rare earth‐based materials. Remarkably, three distinct dynamic afterglow responses, time‐dependent afterglow colors (TDAC), excitation‐dependent afterglow colors (EDAC), and TCAG, were simultaneously exhibited in a single CD system. Using levofloxacin as the luminescent precursor, BUL‐CDs were synthesized with the assistance of boric acid and urea, which demonstrated triple matrix confinement and interfacial effect. The combined matrix confinement and interfacial effects yield ultrabright afterglow and integrate dual afterglow channels, RTP and TADF, within the BUL‐CDs. By exploiting the lifetime contrast between TADF and phosphorescence and their different sensitivities to excitation wavelength and temperature, concurrent TDAC, EDAC, and TCAG were induced in a single material. Further, by combining BUL‐CDs with BL‐CDs (prepared from boric acid and levofloxacin), we constructed a three‐dimensional, stimulus‐responsive color code that changed across time, temperature, and excitation wavelength and spanned the full visible spectrum. This platform enabled high‐capacity, programmable, full‐gamut, and visually intuitive information encryption and display, providing a promising route toward advanced, multidimensional anti‐counterfeiting and secure‐communication technologies.

## 2 Results and Discussion

Employing our previously reported spatial‐confined self‐foaming polymerization methodology [27, 28], the precursor undergoes in‐situ polymerization and subsequent carbonization within the porous foam walls generated by boric acid and urea, yielding the CDs. Mechanistically, boric acid progressively melts upon heating and functions as a solid solvent, while concurrent reaction between urea and boric acid liberates ammonia that drives foaming and the formation of a porous matrix. Within this confined environment, the precursor experiences in‐situ dehydration and carbonization, thereby facilitating the nucleation and growth of CDs.

**FIGURE 1:**
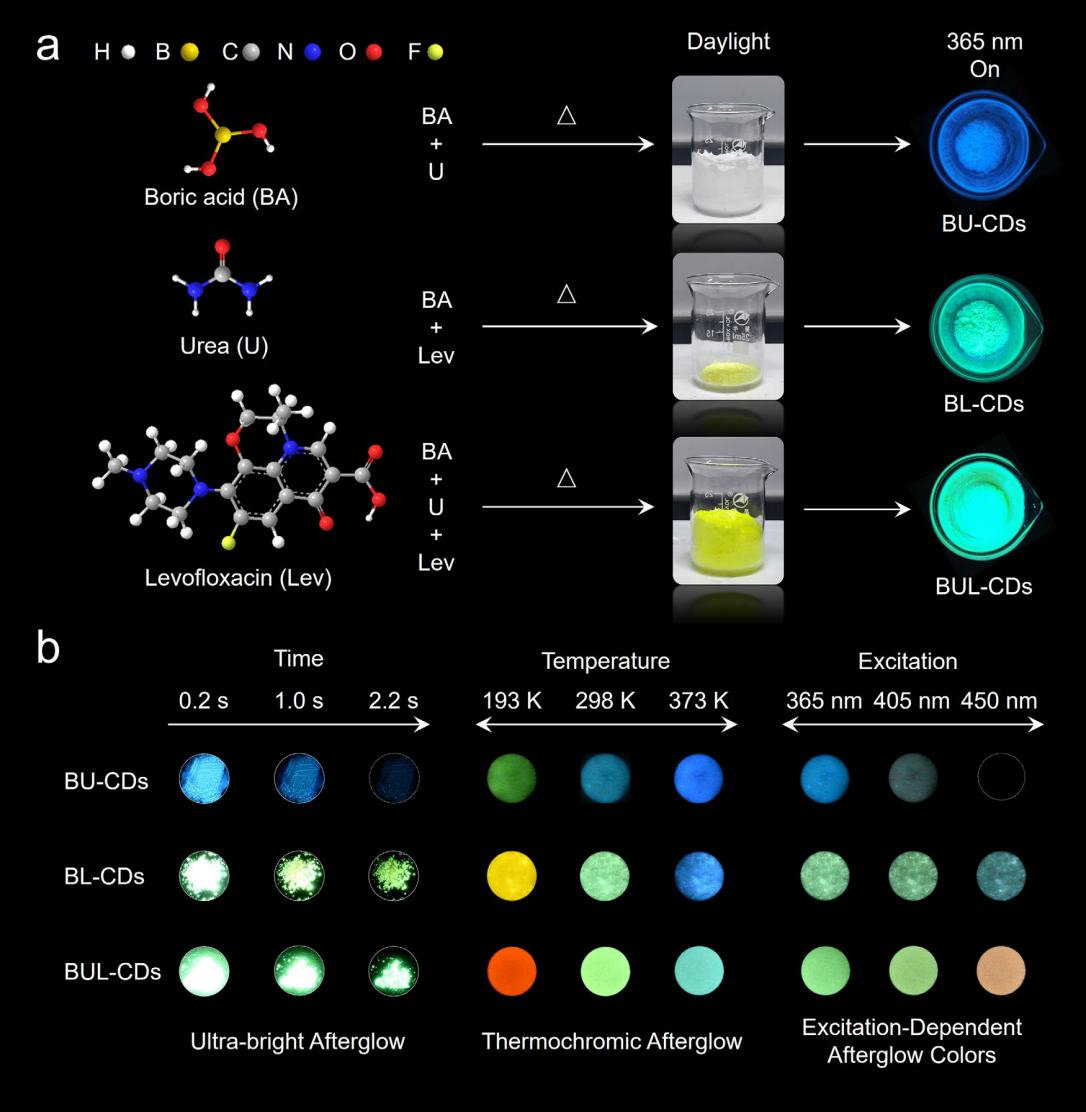
Synthesis and afterglow luminescence of CDs. (a) Schematic diagram of the synthesis of CDs and (b) their afterglow photos at different time points (i), temperature (ii), and excitation wavelength (iii).

The material derived from the boric acid and urea was designated BU‐CDs and exhibited blue photoluminescence (Figure 1a). Incorporation of levofloxacin into the precursor yields BUL‐CDs, which display bright green emission. We initially optimized the ratio of urea to boric acid by preparing a series of precursor mixtures with varying molar ratios (10:1, 8:1, 5:1, 3:1, 2:1, 1:1, 1:2, and 1:5). As shown in Figure S1a, the photoluminescence quantum yield (PLQY) reached its maximum value of 71% at a 1:1 ratio of boric acid to urea. Subsequently, for BUL‐CDs, different amounts of levofloxacin (2.5, 5, 10, 20, and 30 mg) were introduced into the reaction system, yielding PLQYs of 37%, 39%, 61%, 31%, and 33%, respectively (Figure S1b). Based on these results, the optimal precursor composition was determined to be 1 g boric acid, 1 g urea, and 10 mg levofloxacin. As a control, synthesis carried out in the absence of urea produced BL‐CDs, characterized by bright blue‐cyan fluorescence. After the 365 nm UV lamp was switched off, the solid CD powders obtained from all three reactions displayed a pronounced afterglow (Figure 1b). BU‐CDs exhibited a blue afterglow lasting approximately 3 s, whereas BUL‐CDs and BL‐CDs showed slightly longer afterglow of about 6–7 s (Figure S2). Compared with BL‐CDs, BUL‐CDs exhibited a significantly brighter afterglow, suggesting that the incorporation of urea into the precursor played a critical role, as confirmed by subsequent structural characterization.

More importantly, all three types of CDs displayed TCAG behavior. Specifically, as the temperature increased from 193 to 373 K, the afterglow color of BU‐CDs shifted from green to blue, BL‐CDs from yellow to blue, and, remarkably, BUL‐CDs from red to cyan‐green. This indicated that the TCAG of these CDs collectively spanned the entire visible spectrum. The influence of excitation wavelength on the afterglow was further examined. For BU‐CDs, increasing the excitation wavelength resulted in a slight red shift accompanied by a sharp decrease in intensity, with no visible afterglow under 450 nm excitation. BL‐CDs exhibited a slight blue shift under excitation from 365 to 450 nm. In contrast, BUL‐CDs demonstrated pronounced excitation‐dependent color variation: green afterglow under 365 nm excitation (Video S1), yellow‐green at 405 nm, and orange‐red when the excitation shifted to 450 nm (Video S2). Due to their highest afterglow brightness, broader TCAG range, and excitation‐dependent afterglow colors, BUL‐CDs became the primary focus of further investigation.

**FIGURE 2:**
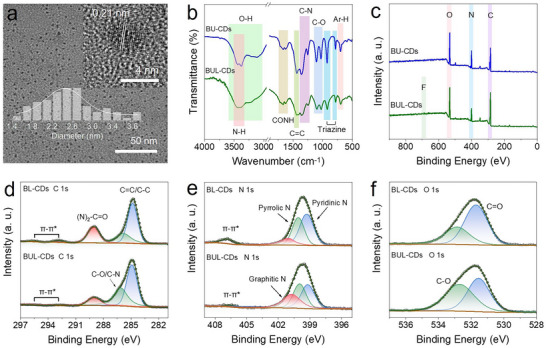
Structural characterization of CDs. a) TEM image of BUL‐CDs, with inset HR‐TEM image and particle size distribution. b) FTIR spectra and c) XPS survey of BU‐CDs and BUL‐CDs. d) C 1s, e) N 1s, and f) O 1s high‐resolution XPS spectra of BU‐CDs and BUL‐CDs.

The morphology and chemical structure of these CDs were comprehensively characterized. Transmission electron microscopy (TEM) analysis revealed that B‐CDs and BUL‐CDs possess uniform particle sizes of approximately 2.7 nm and 2.4 nm, respectively (Figure 2a and S3). In both high‐resolution TEM images, graphite‐like lattice fringes (0.21 nm) were observed, corresponding to the (100) crystal plane of graphene [29]. Fourier transform infrared spectroscopy (FTIR) analysis (Figure 2b) demonstrated that the absorption peak positions of BU‐CDs and BUL‐CDs were generally consistent, indicating their comparable chemical compositions. However, differences in peak intensities were observed. BU‐CDs exhibited stronger absorption bands corresponding to the C = C stretching vibration (∼1436 cm^−1^), C–N vibrations (∼1354 and ∼1251 cm^−1^), the breathing out‐of‐plane vibration of the triazine ring (∼921 cm^−1^), and the sextant out‐of‐plane bending vibration of the triazine ring (∼784 cm^−1^), relative to BUL‐CDs [30]. Notably, the triazine ring vibration appeared between the characteristic peaks of melamine cyanurate (770 cm^−1^) [31] and g‐C_3_N_4_ heptazine (also known as tri‐s‐triazine, ∼810 cm^−1^) [32], suggesting the presence of small triazine ring fragments within the CDs structure, exhibiting intermediate characteristics between the two.

The X‐ray diffraction (XRD) patterns of the crude CDs samples all showed a typical broad diffraction peak of amorphous carbon, accompanied by some characteristic sharp peaks. XRD patterns indicated that BL‐CDs contain only a single extra B_2_O_3_ phase (Figure S4), formed through the dehydration of boric acid during thermal treatment. The diffraction peaks at 14.6°, 27.7° (310), and 30.7° (222) correspond to the crystal planes of B_2_O_3_ (JCPDS No. 06–0297) [33]. In contrast, BU‐CDs and BUL‐CDs exhibit multiple phases in addition to B_2_O_3_. The presence of urea promotes the formation of g‐C_3_N_4_, confirmed by the characteristic peak at ∼26.3°, attributed to the (002) plane of g‐C_3_N_4_ (JCPDS No. 87–1526) [34]. Furthermore, boric acid and urea reacted to form a reported h‐BN‐related intermediate, ammonium bis(biureto)borate NH_4_[B(biu)_2_], where biu = [NH–(C═O)–NH–(C═O)–NH] [35]. The diffraction peaks at 16.5°, 21.3°, 23.0°, 24.9°, and 31.3° were all assigned to NH_4_[B(biu)_2_]. The element B was detected in their X‐ray photoelectron spectroscopy (XPS) (Figure S5–6 and Table S1), and characteristic B–O and B–N bonds were identified in the high‐resolution XPS spectra of B 1s, N 1s, and O 1s. These findings indirectly confirmed the presence of B_2_O_3_ and NH_4_[B(biu)_2_] matrices. Consequently, the BUL‐CDs and BU‐CDs, owing to the supplementary incorporation of urea, comprised three distinct structural matrices, B_2_O_3_, g‐C_3_N_4_, and NH_4_[B(biu)_2_], in contrast to the BL‐CDs system, which was characterized by a single B_2_O_3_ matrix.

Furthermore, matrix‐removed CDs were investigated. XPS survey results of purified BU‐CDs and BUL‐CDs both contained C (∼285 eV), N (∼400 eV), and O (∼532 eV) elements, while BUL‐CDs contained an extra F element (687 eV). However, BUL‐CDs had slightly lower oxygen (19.85%) and nitrogen (21.41%) contents than BU‐CDs (22.09% and 31.91%, respectively), while carbon (58.01%) content was slightly higher than that of BU‐CDs (46.55%). In the high‐resolution XPS spectra, the C 1s spectrum (Figure 2d) could be deconvoluted into three components: C = C/C–C (284.8 eV), C–O/C–N (∼286.0 eV), and N–C = O (289.1 eV), plus a π–π* shake‐up satellite peak (∼292.8 eV) arising from extended delocalized electrons in aromatic rings [36]. BU‐CDs exhibited a higher C = O content (Table S2), with correspondingly larger π–π* areas for aromatic carbons. By contrast, BUL‐CDs showed a higher C–O/C–N ratio, indicating that the addition of levofloxacin generated more defects and reduced π–π stacking in BUL‐CDs. This was attributed to the covalent insertion of the levofloxacin fragment, which increased steric hindrance and interlayer spacing. The N 1s spectrum (Figure 2e) could be deconvoluted into three species: pyridinic N (398.6 eV), pyrrolic N (399.4 eV), and graphitic N (400.2 eV), accompanied by a π–π* transition (406.9 eV) of nitrogen‐containing aromatic rings [37]. BU‐CDs contained higher pyridinic N, pyrrolic N contents, and larger π–π* areas, whereas BUL‐CDs had higher graphitic N content. This suggested that BU‐CDs possessed more g‐C_3_N_4_ triazine ring structures, and the addition of levofloxacin promoted the formation of more graphitic ring‐related structures. In the O 1s region (Figure 2f), two O‐related species were present: C = O (531.5 eV) and C–O (532.7 eV). Compared with BU‐CDs, BUL‐CDs showed lower C = O content and higher C–O content, consistent with the C 1s deconvolution results. Inherited from levofloxacin, the F 1s spectrum of BUL‐CDs (Figure S7) and C‐F bond (687.38 eV) was observed, indicating that the levofloxacin fragment was retained in BUL‐CDs.

Based on the characterization results, it was evident that the carbon cores of both purified BU‐CDs and BUL‐CDs shared similar triazine ring structures, similar to the melamine cyanurate structure. The key difference in BUL‐CDs was that some quinolone fragments derived from levofloxacin were covalently incorporated into the carbon cores due to the competitive reaction involving levofloxacin as a precursor. This incorporation introduced additional defects and increased the interlayer spacing. In the crude CDs samples obtained after the reaction, both BU‐CDs and BUL‐CDs exhibited triple‐matrix confinement, which jointly enhanced the afterglow emission of the CDs. Notably, the triazine ring fragment, which forms the g‐C_3_N_4_ intermediate, also participated in the formation of cores of CDs, enabling a potential unique interfacial contact between the g‐C_3_N_4_ matrix and CDs [38]. For BU‐CDs, the triazine ring fragment itself served as the luminescent center, whereas in BUL‐CDs, the quinolone ring and piperazine ring fragments derived from levofloxacin integrated into the carbon core and established an interfacial interaction with g‐C_3_N_4_, contributing to the bright green afterglow emission of BUL‐CDs.

**FIGURE 3:**
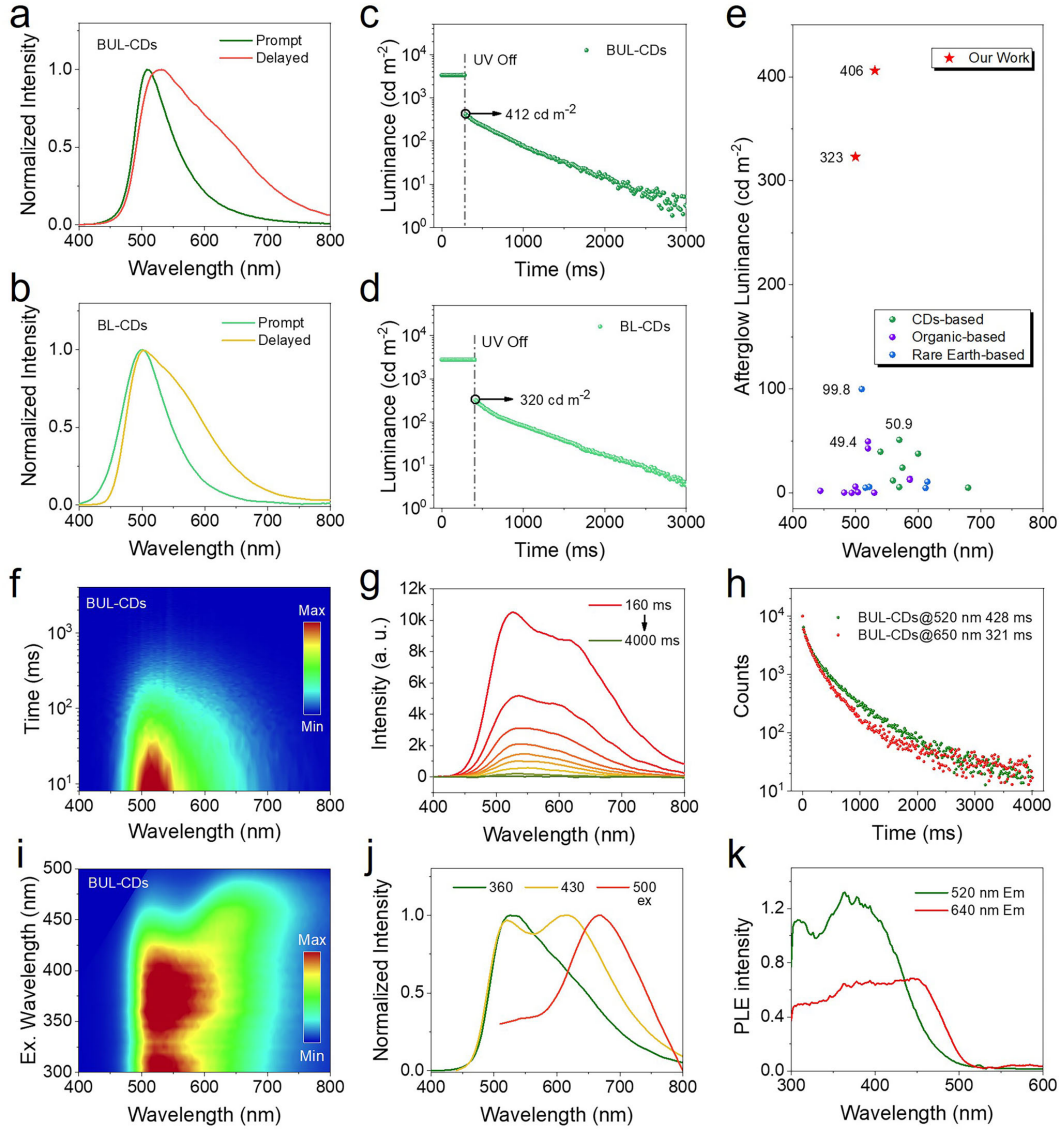
The luminescent properties of CDs. The prompt and delayed PL of a) BUL‐CDs and b) BL‐CDs. Luminance decay curves of c) BUL‐CDs and d) BL‐CDs. Excitation source is a 365 nm UV lamp with a power density of 61 mW cm^−2^. e) Comparison of the initial afterglow luminance of BUL‐CDs and BL‐CDs and other luminescent materials (CDs‐based, organic‐based, and rare earth‐based materials). f) 3D pseudo‐color mapping of the time‐resolved afterglow spectra of BUL‐CDs and g) the afterglow spectra at different decay times from 160 ms to 4000 ms. h) Afterglow lifetime decay curves of BUL‐CDs at 520 nm and 650 nm (excitation wavelength: 365 nm). i) Excitation‐afterglow emission 3D pseudo‐color mapping of BUL‐CDs, j) afterglow emission spectra at different excitation wavelengths, and k) afterglow excitation spectra at 520 nm and 640 nm.

The luminescence properties of the CDs were systematically examined. The prompt emission peak of BUL‐CDs appears at 510 nm (Figure 3a), red‐shifted by approximately 10 nm relative to BL‐CDs (Figure 3b). The delayed emission of BUL‐CDs is centered at 530 nm with a shoulder at 650 nm, whereas BL‐CDs exhibit a delayed peak at 500 nm with a shoulder near 570 nm. The PLQY of BUL‐CDs was 61% (Figure S8), which was higher than that of BL‐CDs (44%). The red‐shift spectra and enhanced PLQY in BUL‐CDs indicated that urea participated in CDs formation, facilitating the development of a more efficient and narrowed bandgap of the triplet state emission. In contrast, BU‐CDs displayed prompt and delayed emission peaks at 393 nm (with a secondary peak at 490 nm) and 470 nm with a PLQY of 71% (Figure S8 and S9). The fluorescence lifetime of BU‐CDs and BUL‐CDs was 2.42 ns and 11.45 ns, respectively (Figure S10). In the absence of levofloxacin, BU‐CDs resulted primarily from limited carbonization and condensation between boric acid and urea (partially forming B_2_O_3_ and g‐C_3_N_4_), yielding CDs with a larger band gap. The ultrabright afterglow of BUL‐CDs and BL‐CDs prompted us to quantify their initial afterglow luminance. Under 365 nm UV irradiation (61 mW cm^−2^), a radiometer recorded luminance before switching off the light (15 ms delayed). By integrating the emission spectra before and after UV cessation, we calculated the initial afterglow luminance via the ratio. As shown in Figures 3c–d, BUL‐CDs and BU‐CDs reach values as high as 412 and 320 cd m^−2^, respectively. After three independent tests, we obtained an average value of 406 ± 5.8 cd m^−2^ for BUL‐CDs and 323 ± 3.0 cd m^−2^ for BU‐CDs, substantially exceeding those reported for CDs‐based and other types of afterglow materials, including organic and rare‐earth‐based systems (Figure 3e and Table S3).

Although BUL‐CDs and BL‐CDs exhibit an initial afterglow intensity far exceeding that of other reported afterglow materials, it should be noted that their afterglow duration remains below 10 s, which constrains their applicability in fields such as delayed illumination. In contrast, rare‐earth‐based afterglow materials typically exhibit persistent luminescence for tens of minutes to several hours [39, 40, 41, 42]. Organic‐based materials with hour‐level afterglow have also been reported [43, 44], and CDs‐based materials with comparable long‐lived persistent luminescence are gradually emerging [13, 45]. Consequently, the development of carbon‐dot‐based afterglow systems that combine ultrahigh initial brightness with persistent luminescence is important in the following research.

Given the dual afterglow emission centers of BUL‐CDs, time‐resolved afterglow spectroscopy was employed to delineate their temporal evolution. As shown in Figures 3f–g, the 520 nm emissive center exhibited slower decay and longer persistence than the 650 nm center. Time‐correlated single‐photon counting measurements yielded afterglow lifetimes of 428 ms and 321 ms at 520 nm and 650 nm, respectively (Table S4). The distinct temporal behaviors of these two emission centers enabled BUL‐CDs to display time‐dependent afterglow colors, further depicted in the following encryption application. We further investigated the luminescence responses of the two centers under varied excitation wavelengths. First, excitation–afterglow emission mapping (Figure 3i) revealed a progressive redshift of the afterglow emission as the excitation wavelength increased from 300 to 500 nm. Afterglow spectra acquired under excitation at 360, 430, and 500 nm (Figure 3j) indicated that the red‐emitting component is increasingly populated with longer excitation wavelengths, producing the observed spectral redshift. Afterglow excitation spectra recorded at 520 nm and 650 nm show that the green‐emitting component predominates below ∼434 nm excitation, whereas the red‐emitting component dominates above ∼434 nm. These differential excitation responses of the two emission centers afford BUL‐CDs excitation‐dependent afterglow color tunability, also illustrated in the following encryption demonstration.

**FIGURE 4:**
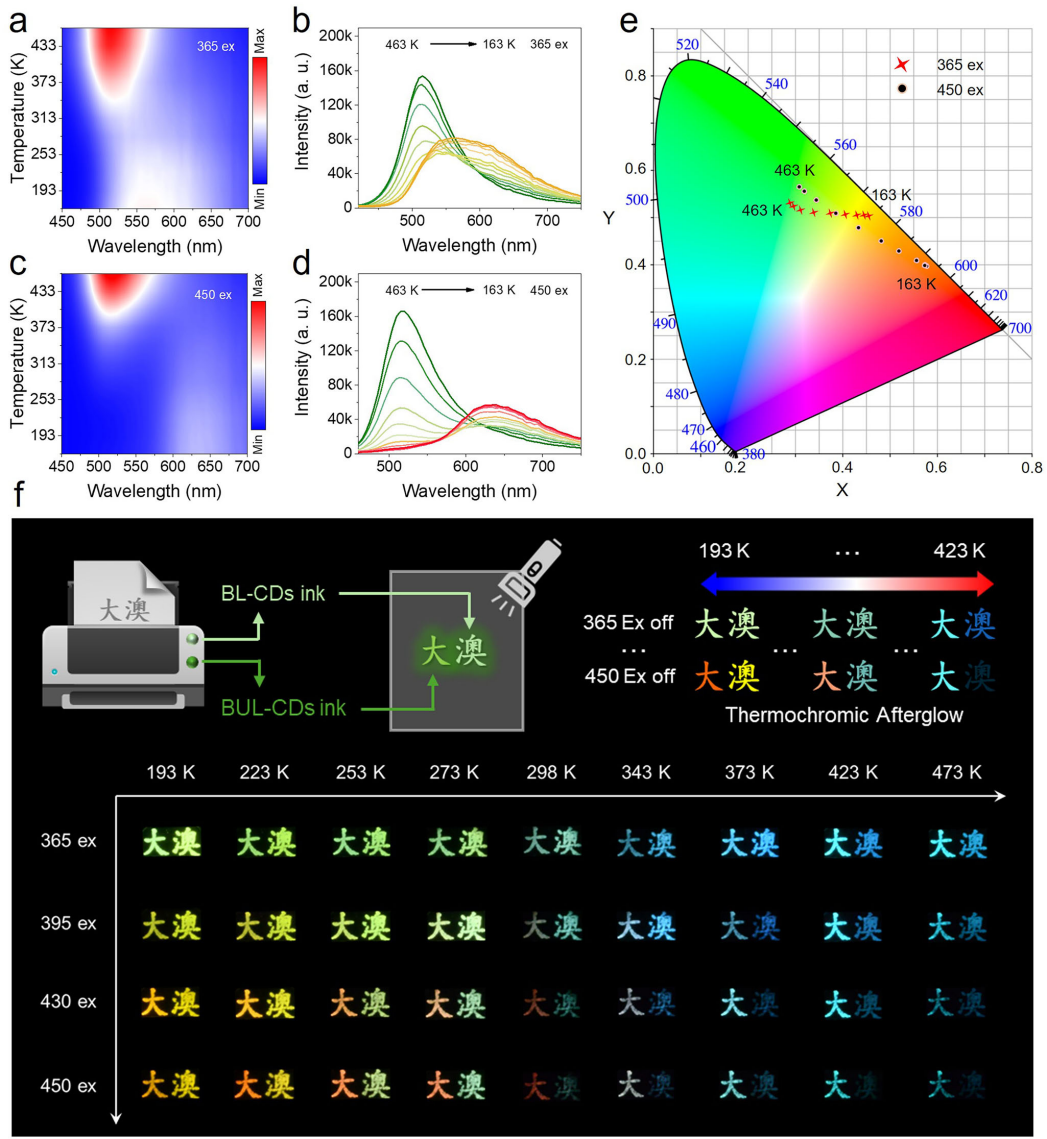
TCAG characteristics of BUL‐CDs at different excitation wavelengths. a) 3D pseudo‐color mapping of temperature‐afterglow emission of BUL‐CDs under 365 nm excitation. b) Afterglow emission spectra of BUL‐CDs at temperatures between 463 and 73 K under 365 nm excitation. c) 3D pseudo‐color mapping of temperature‐afterglow emission under 450 nm excitation. d) Afterglow emission spectra of BUL‐CDs at temperatures between 463 and 73 K under 450 nm excitation. e) Color coordinates of afterglow emission spectra of BUL‐CDs at different temperatures (red stars: 365 nm excitation, black dots: 450 nm excitation). f) Schematic diagram and actual pattern demonstration of TCAG pattern designs using BL‐CDs and BUL‐CDs inks under different excitation wavelengths.

Next, we sought to identify the two afterglow emission centers. To this end, temperature‐dependent afterglow spectra were first examined. Based on the previously discussed excitation‐dependent color characteristics, afterglow spectra at various temperatures were recorded under excitation at 365 nm and 450 nm. As shown in Figures 4a–b, at high temperature (463 K), BUL‐CDs exhibited a green afterglow with a dominant peak at 520 nm, whereas the emission centered at 640 nm gradually intensified as the temperature decreased. At low temperature, the afterglow spectrum was clearly a superposition of two components (Figures 4c–d). And the *ΔE_st_* of BUL‐CDs was calculated as 0.45 eV. Notably, excitation at 365 nm could simultaneously activate both emission centers at low temperature, making them difficult to distinguish. In contrast, under 450 nm excitation, the green afterglow observed at high temperature (463 K) progressively weakened with decreasing temperature, while the red afterglow at low temperature (163 K) became more pronounced. The former (green emission center) aligns with the characteristic behavior of TADF, whose intensity increases with temperature, whereas the latter (red emission center) corresponds to phosphorescence, which strengthens as temperature decreases. Furthermore, the CIE 1931 color coordinates (Figure 4e) of BUL‐CDs excited at 365 nm shift from yellow to green as the temperature rises from 163 to 463 K, while under 450 nm excitation, they transitioned from red to green.

Inspired by their TCAG properties, we first explored their use as inks for paper‐based anti‐counterfeiting labels. Aqueous solutions of BL‐CDs and BUL‐CDs were used to print the Chinese characters “澳” (Ao) and “大” (Da), the abbreviation for the University of Macau, on filter paper. Afterglow images were recorded under different excitation wavelengths (365, 395, 430, and 450 nm) and across a temperature range of 193–473 K (Figure 4f ). Under 365 nm excitation, the character “澳” exhibited yellow phosphorescence at low temperature that converted to blue TADF at high temperature, whereas “大” changed from green phosphorescence at low temperature to cyan TADF at high temperature. As the excitation wavelength increased to 450 nm, the change in “澳” became negligible aside from a slight decrease in afterglow brightness; by contrast, “大” showed progressively red‐shifted afterglow at low temperatures while remaining cyan at high temperatures, albeit with a modest reduction in intensity.

**FIGURE 5:**
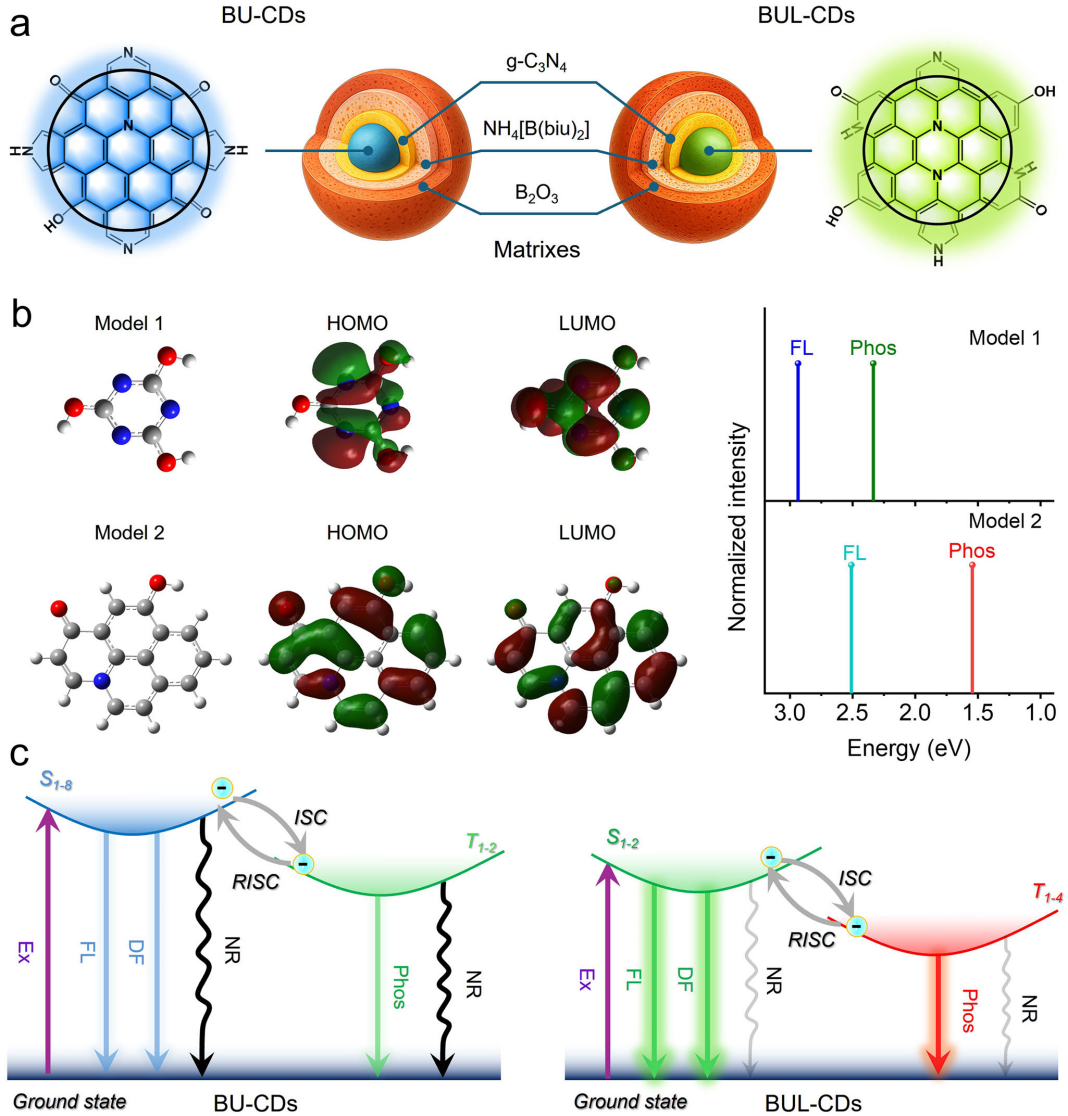
Mechanism of afterglow of BU‐CDs and BUL‐CDs. a) Proposed CDs structures along with their triplet matrices. b) Fluorescence and phosphorescence emission energies were obtained by time‐dependent DFT calculations based on the possible structures of BU‐CDs and BUL‐CDs luminescent units. c) Jablonski diagram of delayed fluorescence and phosphorescence transition processes in BU‐CDs (left) and BUL‐CDs (right). Ex: Excitation; FL: Fluorescence; DF: Delayed Fluorescence; NR: Non‐radiative; Phos: Phosphorescence.

The mechanism of afterglow luminescence of CDs was further investigated. Structural characterization revealed that BU‐CDs were enriched in pyridinic and pyrrolic nitrogen, whereas BUL‐CDs contain a higher proportion of graphitic nitrogen. Based on these distinctions, schematic structures were proposed (Figure 5a). Both BU‐CDs and BUL‐CDs feature a triple‐matrix confinement comprising: (i) g‐C_3_N_4_ derived from urea at elevated temperature, (ii) B_2_O_3_ generated from boric acid, and (iii) NH_4_[B(biu)_2_] species formed from boric acid and urea, serving as intermediates in BN formation. Because NH_4_[B(biu)_2_] contains B–N bonds, it acts as a chemical bridge between the g‐C_3_N_4_ and B_2_O_3_ layers, tightly coupling the matrices and significantly reinforcing the confinement. Furthermore, considering that the afterglow of BUL‐CDs can be effectively excited by the blue emission mainly from g‐C_3_N_4_ in the matrix (Figure 3i and S9a), an afterglow energy resonance transfer (RET) process occurs between the matrix and the CDs. This synergistic effect of enhanced confinement and RET is manifested in the markedly higher afterglow intensity of BUL‐CDs compared with BL‐CDs, which possess only a single B_2_O_3_ matrix (Figure 3e).

To elucidate the emissive origins, density functional theory (DFT) calculations were performed on minimal luminescent units representative of BU‐CDs and BUL‐CDs (Figure 5b). Given that the blue emission of BU‐CDs originates from urea‐derived triazine‐related species, cyanuric acid (Model 1) was rationally selected as the smallest plausible luminescent unit. Time‐dependent DFT (TD‐DFT) predicted fluorescence and phosphorescence emission energies of 2.93 eV and 2.33 eV, which were consistent with experimental results in Figure 1b. For BUL‐CDs, emission was attributed to the quinolone segment of levofloxacin. Because XPS analysis showed comparable amounts of C = O and C–O groups, Model 2 incorporated a quinolone core bearing a hydroxyl substituent.TD‐DFT for Model 2 illustrated pronounced redshifts in both fluorescence and phosphorescence relative to Model 1 (by 2.51 eV and 1.54 eV, respectively), consistent with the experimental spectra shift trend. Furthermore, we calculated the energy level transitions and spin‐orbit coupling (SOC) constants for Models 1 and 2 (Figure S11). The transition analysis [46, 47] reveals that the S_0_‐S_8_ transition in Model 1, with the highest oscillator intensity (f = 0.25990, Table S5), is the most likely transition path during photoexcitation. The excited electron in the S_8_ state then reaches the S_1_ state via internal conversion (IC). Subsequently, one possible ISC channel (S_1_‐T_2_) with a relatively large SOC constant (ξ = 11.24 cm^−1^) was found (Figure S11a), which then reaches T_1_ via IC and emits phosphorescence. In Model 2, the electronic excitation path is S_0_‐S_2_ (f = 0.19180, Table S6). It is worth noting that the SOC of S_2_‐T_4_ is as high as 14.11 cm^−1^, which is the main ISC and RISC channel (Figure S11b).

Based on the experimental and simulation findings, the luminescence mechanisms illustrated in Figure 5c are proposed. Although BU‐CDs also benefit from triple‐matrix confinement, the intrinsically low luminescence efficiency of g‐C_3_N_4_ leads to dominant non‐radiative decay pathways, resulting in a low radiative fraction and weak fluorescence and afterglow. In contrast, quinolone units in BUL‐CDs exhibit high luminescence efficiency with minimal non‐radiative losses; under triple‐matrix co‐confinement, they produce an ultra‐bright afterglow (up to 406 cd·m^−2^). At room temperature, BUL‐CDs undergo simultaneous ISC and RISC, generating a yellow–green afterglow that represents a superposition of green TADF and red phosphorescence. The distinct lifetimes of these two emissive channels enable TDPC. Their opposite temperature sensitivities give rise to TCAG. Furthermore, because TADF and phosphorescence are preferentially excited by different wavelengths (short‐wavelength excitation, e.g., 365 nm, favors TADF, whereas longer‐wavelength excitation, e.g., 450 nm, preferentially excites phosphorescence), EDPC is also achieved. In summary, the quinolone fragment of levofloxacin, confined within the triple‐matrix architecture, accounts for the ultra‐bright, dual‐mode (TADF and phosphorescence) afterglow of BUL‐CDs, which can be modulated by time, temperature, and excitation wavelength to realize TDPC, TCAG, and EDPC—features that provide a robust platform for advanced encryption schemes.

**FIGURE 6:**
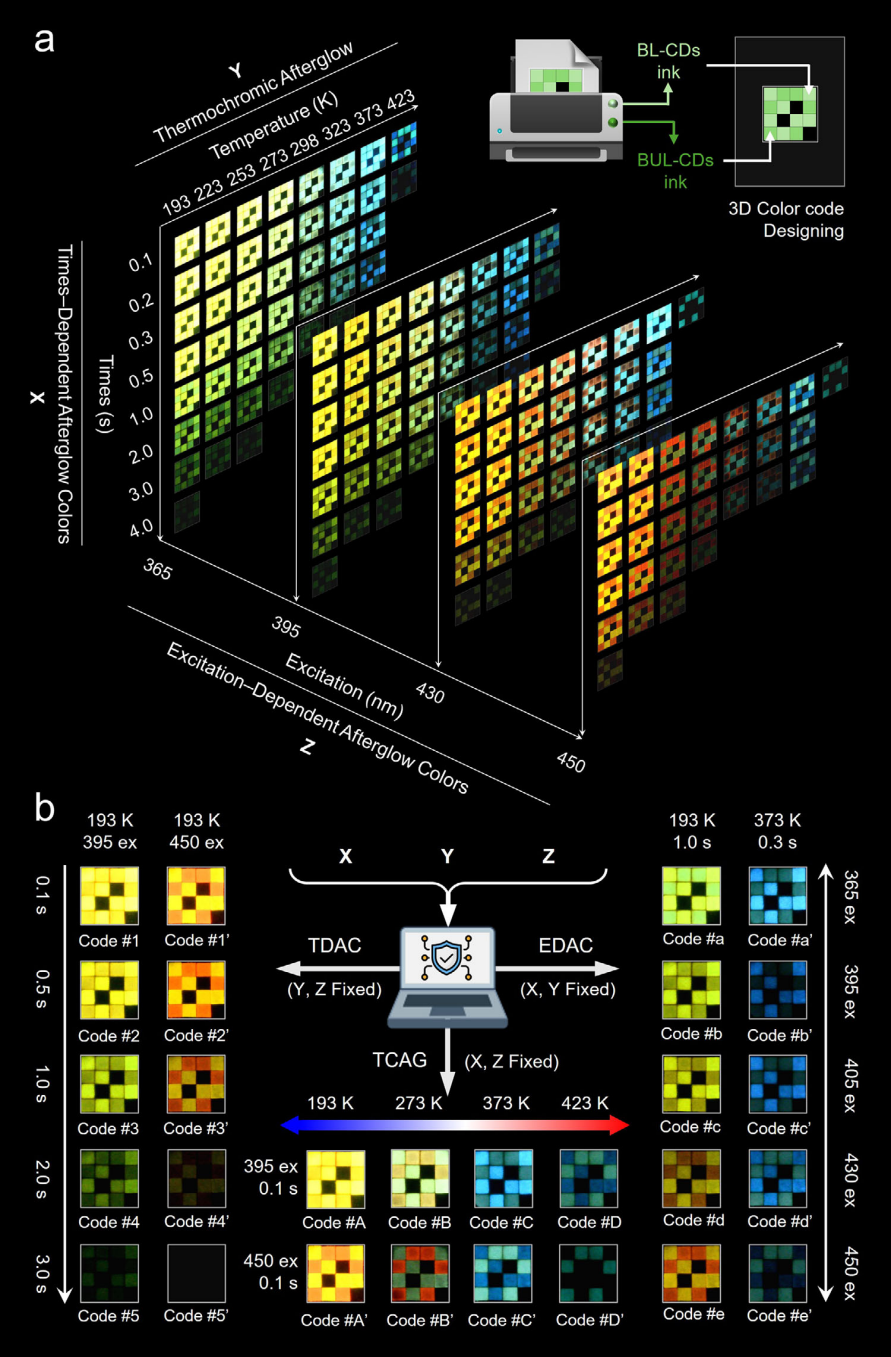
Color codes were designed using BL‐CDs and BUL‐CDs inks for advanced information encryption. a) The designed code demonstrates 3D encoding capabilities at different times, temperatures, and excitation wavelengths. b) Simultaneous display of time‐dependent afterglow color, thermochromic afterglow, and excitation‐dependent afterglow color.

The structure‐luminescence‐property study above confirmed that BUL‐CDs possessed unique triple‐response afterglow discoloration properties: TDAC, EDAC, and TCAG, which greatly supported our determination to apply them to high‐level information encryption. The color codes were designed, as shown in Figure 6a (top right). All the small squares were divided into three regions, marked with BL‐CDs ink and BUL‐CDs ink respectively, and a blank area (black block). By creatively introducing three variables (X: time, Y: temperature, Z: excitation wavelength), full‐color afterglow codes could be generated in three‐dimensional space, as depicted in Figure 6a. Compared with single‐response color‐changing afterglow codes, the three‐dimensional variable response greatly increased the information storage capacity. When the (X, Y, Z) key was stored in the computer to build a key database, entering any (x, y, z) password would return a color code, theoretically yielding X×Y×Z combinations, far exceeding the single‐response afterglow discoloration encoding.

As a simplified demonstration, fixing any two variables (X, Y, Z) yielded a series of color‐changing codes, as shown in Figure 6b. For example, fixing Y and Z at 193 K and 395 nm excitation, we obtained a series of time‐dependent afterglow color codes. It is worth noting that TDAC could be constructed not only by the lifetime of the two afterglow centers of BUL‐CDs (changing from yellow to green afterglow), but also by the difference in afterglow decay between BUL‐CDs and BL‐CDs. We collected color codes at five time points: 0.1 s, 0.5 s, 1.0 s, 2.0 s, and 3.0 s, naming them code #1–5, respectively. Clearly, each code displayed a unique color and carried different encryption information. Similarly, fixing Y and Z at 193 K and 450 nm excitation yielded a series of color codes #1’–5’ representing different encryption information. When we fixed X and Y at 193 K and 1.0 s, a series of afterglow color codes #a–e appeared, exhibiting excitation‐wavelength dependence. Specifically, the color squares marked with BL‐CDs ink remained yellow phosphorescence, while the color squares marked with BUL‐CDs ink gradually changed from green to red as the excitation wavelength increased, exhibiting a fascinating transition from code #a to code #e and storing different encrypted information. Similarly, when X and Y were fixed at 373 K and 0.3 s, excitation‐dependent afterglow color codes #a’–e’ dominated by TADF appeared.

Further, when X and Z were fixed, the TCAG color codes were realized. At X = 395 nm excitation, Z = 0.1 s, the BL‐CDs markings in the afterglow color code gradually changed from yellow to green, then to cyan, and finally to blue as the temperature increased (193 K–423 K), while the BUL‐CDs markings changed from yellow to yellowish‐green and then to green, forming codes #A–D. Similarly, at X = 450 nm excitation, Z = 0.1 s, as the temperature increased, the BL‐CDs markings gradually changed from yellow to green, then to blue, and finally disappeared, while the BUL‐CDs markings changed from bright red to dark red, then to bright green, and finally to dark green. These combinations of TCAG color blocks constituted codes #A’–D’, carrying unique secret information. It's worth noting that increasing the truncation density of any variable would yield more afterglow color codes, and with high‐resolution reading devices, this would greatly expand the number and information capacity of the afterglow color codes.

However, it is noteworthy that the introduction of excitation, time, and temperature variables enriches the diversity of information encryption, yet simultaneously increases the instability of the encryption system. As shown in the lower right corner of each excitation in Figure 6a, no actual 3D code patterns are present, indicating that under high‐temperature conditions and prolonged delay, the database would fail to return valid information. For instance, when the input is set as (X = 1.0, Y = 423, Z = arbitrary), the database output is essentially blank. Therefore, future work should focus on developing stable long‐persistence emission CDs under high temperatures to improve the stability of the afterglow response under multivariate stimuli.

## 3 Conclusion

In summary, leveraging triple‐matrix confinement, levofloxacin‐derived BUL‐CDs exhibited an exceptionally high initial afterglow brightness of 406 cd m^−2^, far surpassing that of other luminescent materials. More importantly, they simultaneously achieved triple‐responsive‐dependent afterglow colors: TDAC, EDAC, and TCAG, within a single material. During the solid‐state self‐foaming process, boric acid and urea formed three matrices—B_2_O_3_, g‐C_3_N_4_, and additional NH_4_[B(biu)_2_], while urea also contributed to the formation of the carbon core. Structural characterization revealed that triazine ring fragments constituted the carbon core framework of the CDs. Compared with BL‐CDs prepared without urea, the incorporation of urea significantly enhanced both PLQY and initial afterglow brightness in BUL‐CDs, underscoring its critical role. Benefiting from dual‐mode TADF and phosphorescence emission, BUL‐CDs exhibited green TADF and red phosphorescence transitions under diverse external stimuli. As a proof of concept, a three‐dimensional color code constructed from BL‐CDs and BUL‐CDs demonstrated temperature‐, excitation wavelength‐, and time‐dependent panchromatic afterglow color changes. By integrating multiple variables and color variations, this system achieved a substantially higher information storage capacity than conventional black‐and‐white QR codes and single‐variable color codes. Our CDs‐based, multi‐stimulus‐responsive color‐changing afterglow material provides new insights for advancing carbon‐based luminescent materials and developing next‐generation information encryption technologies.

## Conflicts of Interest

The authors declare no conflicts of interest.

## Supporting information




**Supporting File 1**:The authors have cited additional references within the Supporting Information [24, 1–18].

**Supporting File 2**: anie71585‐sup‐0002‐Video S1‐365 EX 93K‐423K‐1080p.mp4.

**Supporting File 3**: anie71585‐sup‐0003‐Video S2‐450 EX 93K‐423K‐1080p.mp4.

## Data Availability

The data that support the findings of this study are available from the corresponding author upon reasonable request.
