# Supplementary material for: Triple Matrix Confinement‐Induced Ultrabright Afterglow From Carbon Dots With Multivariate Responsive Afterglow Colors for Advanced Dynamic Information Encryption
Source: Angew Chem Int Ed Engl. 2026 Mar 2;65(16):e8266335. doi: 10.1002/anie.8266335 (PMC13080412; doi:10.1002/anie.8266335)
Supplement: Supplementary file 1 — Supporting File 1:The authors have cited additional references within the Supporting Information [24, 1–18]. [file ANIE-65-e8266335-s001.docx]

*Supporting Information for*

**Triple Matrix Confinement-Induced Ultrabright Afterglow from Carbon Dots with Multivariate Responsive Afterglow Colors for Advanced Dynamic Information Encryption**

Yupeng Liu^[a,b]^, Yiming Hao^[c]^, Yi Li^[d]^, Xiaofan Xia^[e]^, Xichang Ou^[f]^, Xue Wu^[a]^, Jun Wu^[a]^, Ruifeng Zheng^[a]^, Dongbo Guo^[g]^, Shi Chen^[a]^, Jing Li^[f]^, Jinyang Zhu^[e]^, Qijun Li^[h]^*, Songnan Qu^[a,b,c]^*

[a] Y. Liu, X. Wu, J. Wu, R. Zhang, S. Chen, S. Qu
Joint Key Laboratory of Ministry of Education, Institute of Applied Physics and Materials Engineering (IAPME), University of Macau, Taipa, Macau SAR, 999067 China

[b] Y. Liu, S. Qu

Zhuhai UM Science and Technology Research Institute, University of Macau, Taipa, Macau SAR, 999067 China

[c] Y. Hao, S. Qu
Department of Physics and Chemistry, Faculty of Science and Technology, University of Macau, Taipa, Macau SAR, 999067 China

[d] Y. Li
School of Mechanical Engineering, Jiangsu University, Zhenjiang, 212013 China

[e] X. Xia, J. Zhu
State Centre for International Cooperation on Designer Low-Carbon & Environmental Materials, School of Materials Science and Engineering, Zhengzhou University, Zhengzhou, 450001 China

[f] X. Ou, J. Li
School of Chemical Engineering and Light Industry, Guangdong University of Technology, Guangzhou, China

[g] D. Guo
State key laboratory of digital medical engineering, Sanya Research Institute of Hainan University, School of Biomedical Engineering, Hainan University, Sanya, 572025 China

[h] Q. Li
School of Mechanical Engineering, Institute of Technology for Carbon Neutralization, Yangzhou University, Yangzhou, 225009 China

Email: [liqijun@yzu.edu.cn](mailto:liqijun@yzu.edu.cn) (Q. Li); [songnanqu@um.edu.mo](mailto:songnanqu@um.edu.mo) (S. Qu)

**Table of Contents**

**Experimental Section**

**General method**

**Preparation of CDs ink**

**Time-dependent Density Functional Theory Calculations**

**Figure S1.** Optimization of precursor ratio.

**Figure S2.** Afterglow photos of three type of CDs after 365 nm excitation off.

**Figure S3.** a) TEM image of BU-CDs, with inset HR-TEM image. b) Particle size distribution.

**Figure S4.** Xrd pattern of crude CDs.

**Figure S5.** XPS of crude BU-CDs sample.

**Figure S6.** XPS of crude BUL-CDs sample.

**Figure S7.** F 1s XPS of crude BUL-CDs sample.

**Figure S8.** PLQY of a) BU-CDs, b) BL-CDs, c) BUL-CDs.

**Figure S9.** a) Normalized PL and afterglow spectra of BU-CDs under 365 nm excitation. b) Afterglow decay of BU-CDs under 365 nm excitation.

**Figure S10.** The fluorescence lifetime of BU-CDs and BUL-CDs.

**Figure S11.** The calculated energy-level difference diagrams and spin-orbit coupling constants.

**Table S1.** XPS contents of CDs before dialysis.

**Table S2.** XPS contents of CDs after dialysis.

**Table S3.** Comparison of the reported initial afterglow brightness of luminescent materials.

**Table S4.** Afterglow lifetime of CDs.

**Table S5.** Energy level transition information of S_0_-S_n_ (n=1-10) in Model 1.

**Table S6.** Energy level transition information of S_0_-S_n_ (n=1-10) in Model 2.

**Reference.**

**Experimental Section**

**Materials**

Levofloxacin was purchased from Macklin Pharmaceutical Co., Ltd. (Shanghai). Boric acid and urea were purchased from Aladdin Reagent Co., Ltd. (Shanghai). All reagents were used directly without any additional purification. Ultrapure water was used for all experiments.

**Synthesis of CDs**

The synthesis of BU-CDs based on spatially confined self-foaming technology. In detail, Urea (1g) and Boric acid (1g) were mixed evenly in an open beaker before being put in a constant oven and heated at 200 °C for 5h. After cooling to room temperature, the obtained white porous solid was dissolved in ultrapure water and dialyzed for 24 h with a dialysis bag of 500 Da cutoff. Then the purified solution was freeze-dried to obtain a white solid. The synthesis of BUL-CDs follows the same procedures, only with the addition of 10 mg levofloxacin in the beaker before heating. The obtained crude porous solid was light yellow and yielded a brown solid after dialysis. The BL-CDs were synthesis following the process in the previous report.^[1]^

**General Method**

TEM (FEI Tecnai-G2-F30 operated at 200 kV) and field emission scanning electron microscopy (FE-SEM, Carl Zeiss, Sigma) were used for morphological studies. TEM samples are prepared by dilute aqueous solution after dialysis. Detailed, the dilute aqueous solution after dialysis was dripped onto a copper mesh covered with ultra-thin supporting carbon film and then vacuum-dried at room temperature for 6 h. The crystal structure was measured by X-ray diffraction (XRD, Rigaku, Smart lab). NMR spectra were recorded using a Bruker AV-400 instrument (Bruker, Germany). The UV–vis absorption spectra were collected on a UV–vis–NIR spectrophotometer (Jasco V-770), and the PL spectra, PLQY, and PL decay were collected at room temperature on an Edinburgh FS5 spectrophotometer. The time-resolved phosphorescence spectra were performed at room temperature by Edinburgh FLS5 spectrofluorometer equipped with a microsecond flash-lamp (μF900) with the following settings: total decay time, 4 s; delay time, 0.1 ms; gate time, 1 ms. The PL lifetime was re-confirmed on time-resolved confocal microscopy (PicoQuant Microtime 200). The INSTEC HCS421V hot and cold Stage with a temperature range from -190 °C to 400 °C is for controlling the temperature of samples in measurement. Optically pumped PL spectra were collected by a Princeton spectrometer (Acton SpectraPro SP-2300, Teledyne Princeton Instruments, USA). TRPL spectra and images were measured with a Hamamatsu streak camera system which has an ultimate temporal resolution of 1 ps. TA measurements were performed using an Ultrafast System HELIOS spectrometer with a nondegenerate pump-probe configuration. The broadband probe pulses were generated by focusing a small portion of the fundamental 800 nm laser pulses into a CaF2 plate. The fundamental-frequency 800 nm femtosecond laser pulse was output from the Coherent Astrella-1K-F Ultrafast Ti: Sapphire Amplifier (<100 fs, 1 kHz). The excitation pump pulses used in the TA, PL, and TRPL measurements were generated from a Light Conversion TOPAS-C optical parametric amplifier. The photos and videos were recorded by a smartphone (Redmi K50) or a Nikon digital camera (D7500).

**Preparation of CDs ink**

CDs was dispersed in deionized water to form a uniform solution (1 mg mL^−1^, respectively). The CDs solution was directly used as ink by injecting it into a vacant cartridge of a commercial inkjet printer (EPSON L3150 printer).

**Time-dependent Density Functional Theory Calculations**

The B3LYP density functional method was employed in this work to carry out all computations. The 6–31G(d) basis set was used for the atoms in the geometry optimization. Vibrational frequency analyses at the same level of theory were performed on all the optimized structures to characterize the stationary points as local minima. After artificially assigning an oscillator intensity to the calculated phosphorescence excitation energy, the phosphorescence spectrum is obtained through Gaussian broadening. All calculations were performed in the gas phase. The Gaussian 16 and GaussView 6 suit was used throughout this study. The energy-level difference diagrams and spin-orbit coupling constant matrix were calculated using the ORCA software package (version 4.2.0). Energy level transition information was analyzed using Multiwfn (version 3.8) software.^[2, 3]^ The calculations of this work were performed in part at the high-performance computing cluster (HPCC) supported by the information and communication technology office (ICTO) of the University of Macau.


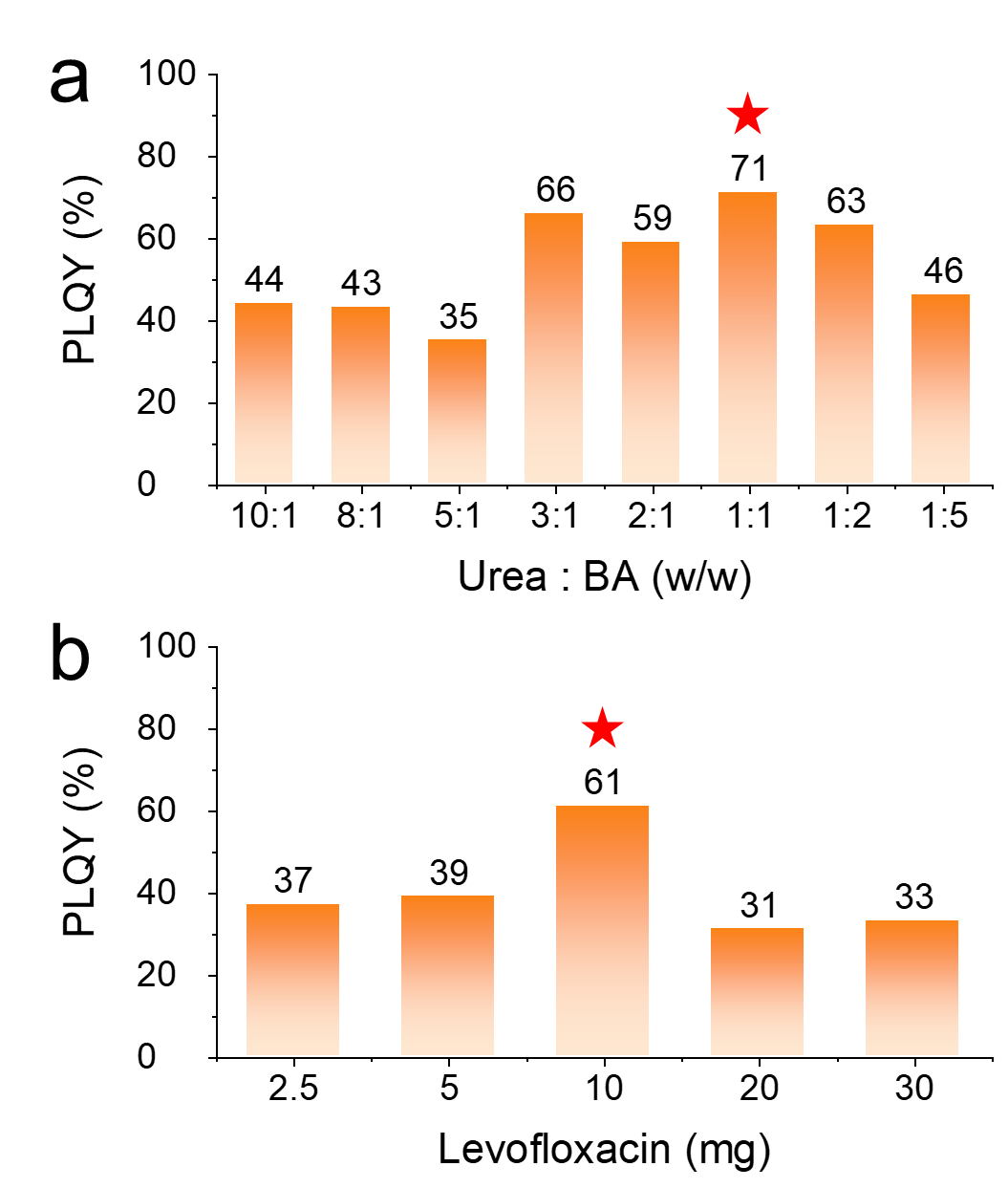


**Figure S1.** Optimization of precursor ratio. a) Optimization of precursor ratios. a) PLQY of samples prepared with different urea and boric acid ratios. b) PLQY of samples prepared with different amounts of levofloxacin added.


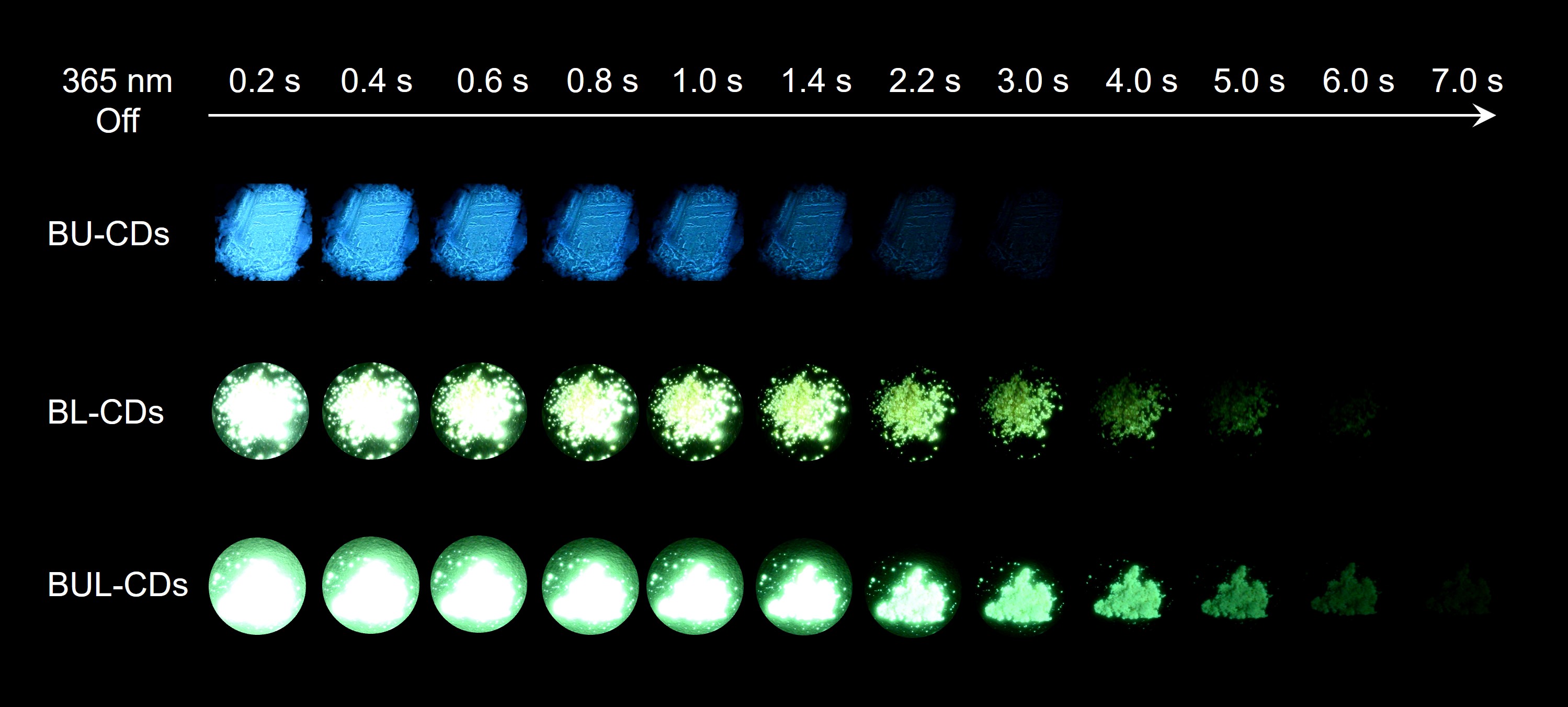


**Figure S2.** Afterglow photos of three type of CDs after 365 nm excitation off.


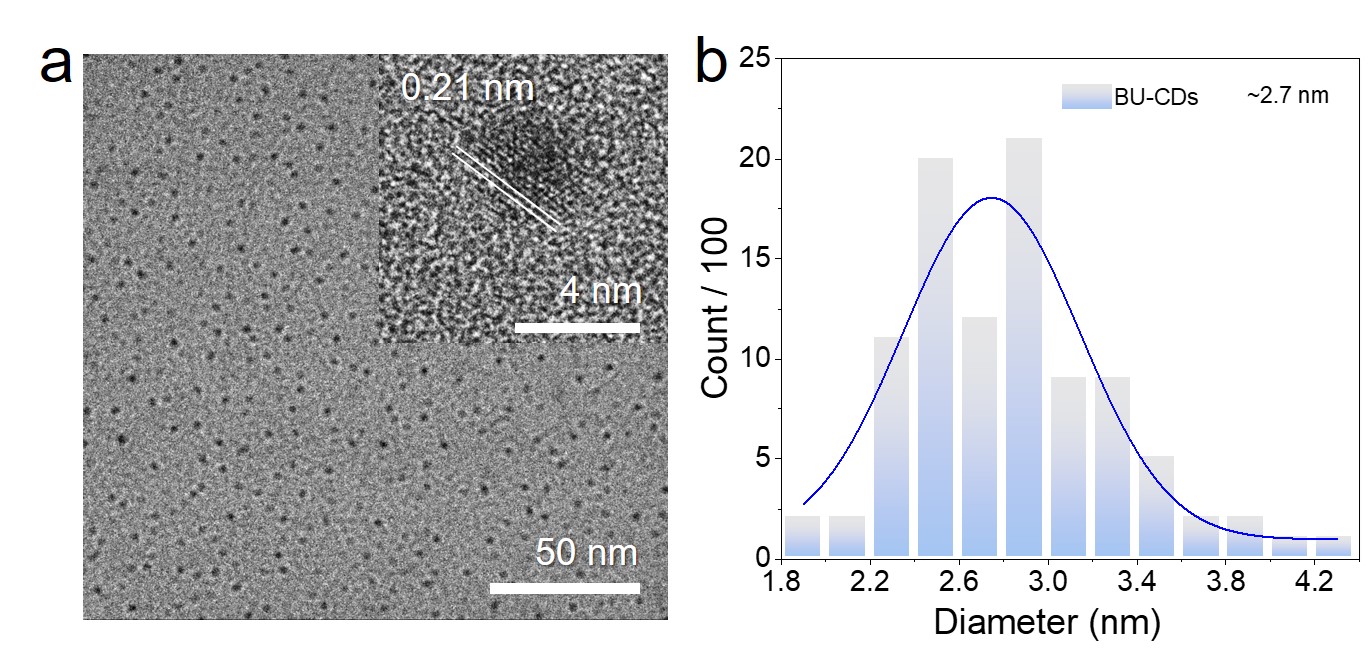


**Figure S3.** a) TEM image of BU-CDs, with inset HR-TEM image. b) Particle size distribution.


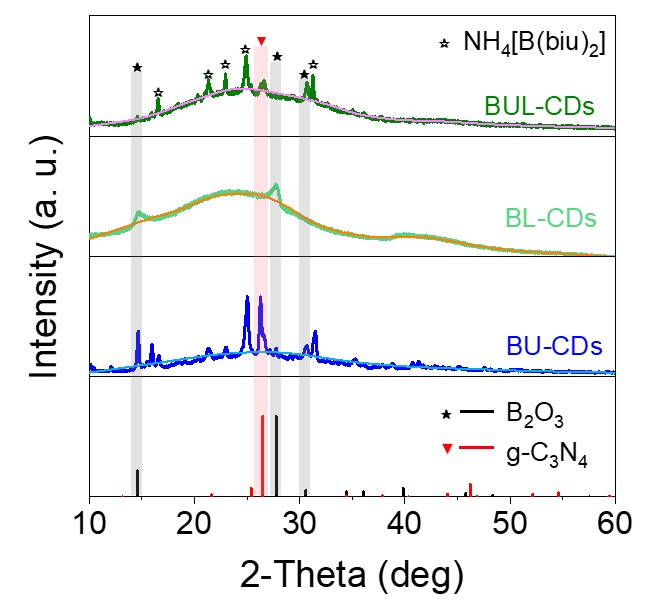


**Figure S4.** Xrd pattern of crude CDs.


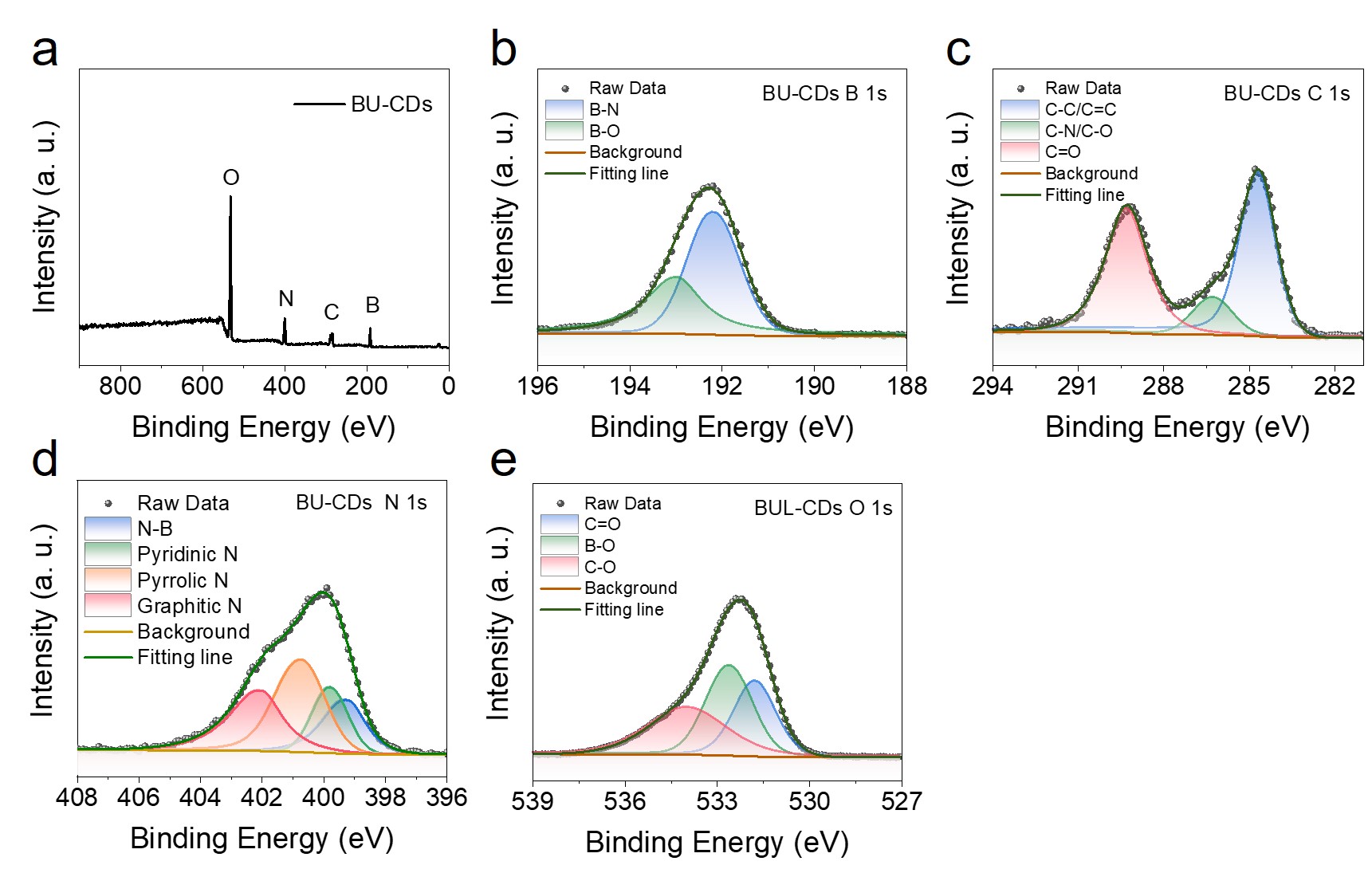


**Figure S5.** XPS of crude BU-CDs sample.


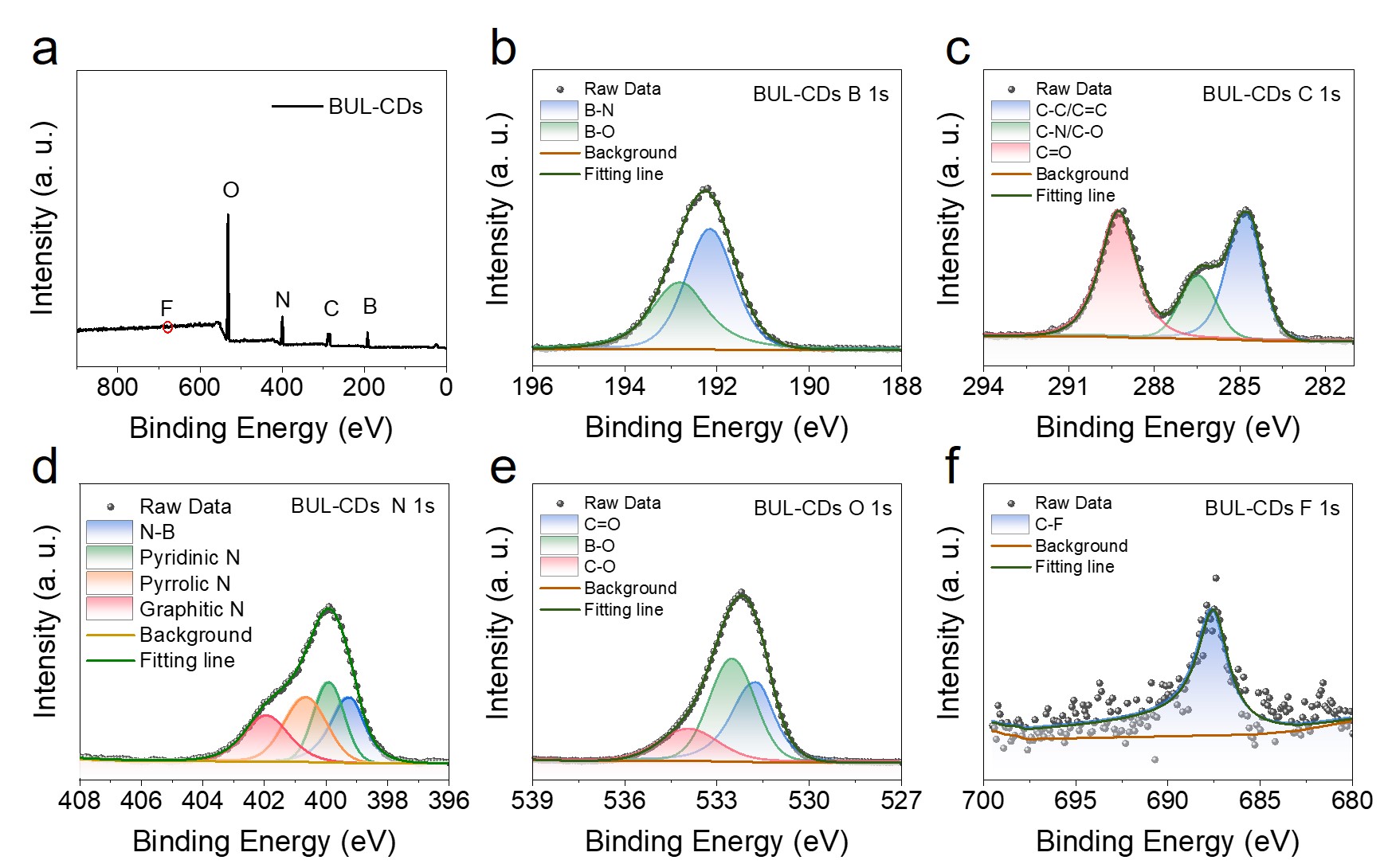


**Figure S6.** XPS of crude BUL-CDs sample.


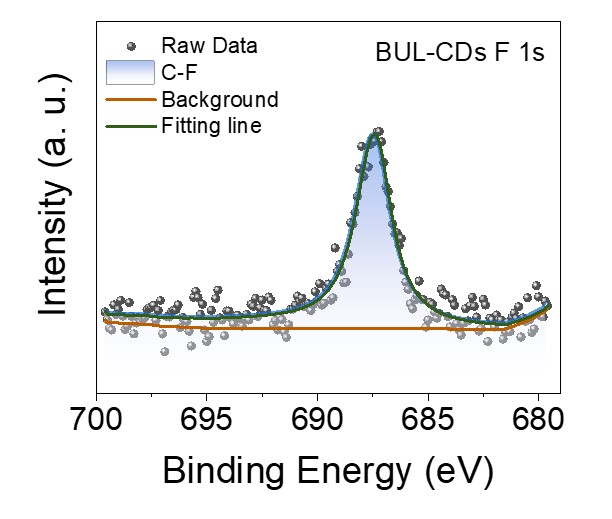


**Figure S7.** F 1s XPS of crude BUL-CDs sample.


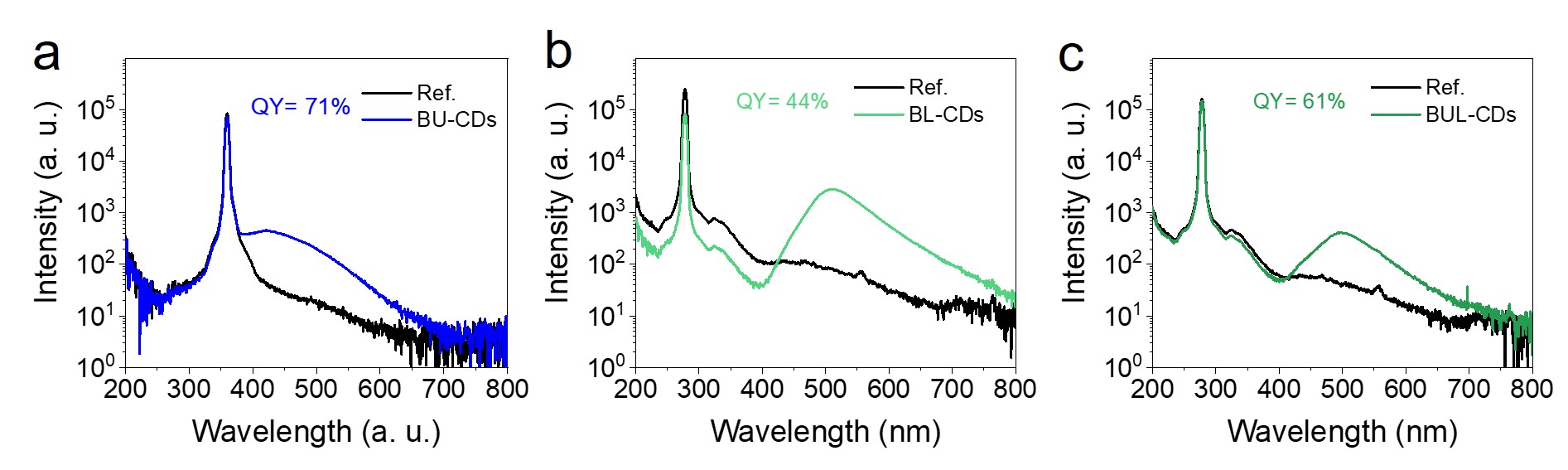


**Figure S8.** PLQY of a) BU-CDs, b) BL-CDs, c) BUL-CDs.


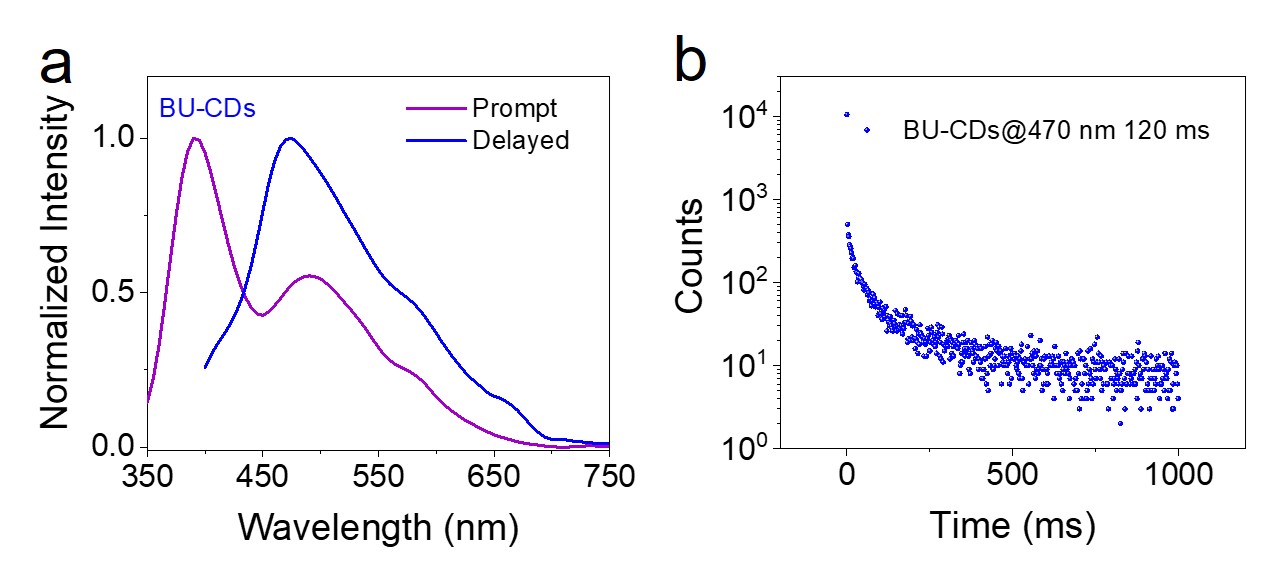


**Figure S9.** a) Normalized PL and afterglow spectra of BU-CDs under 365 nm excitation. b) Afterglow decay of BU-CDs under 365 nm excitation.

**Figure S10.** The fluorescence lifetime of BU-CDs and BUL-CDs.

**
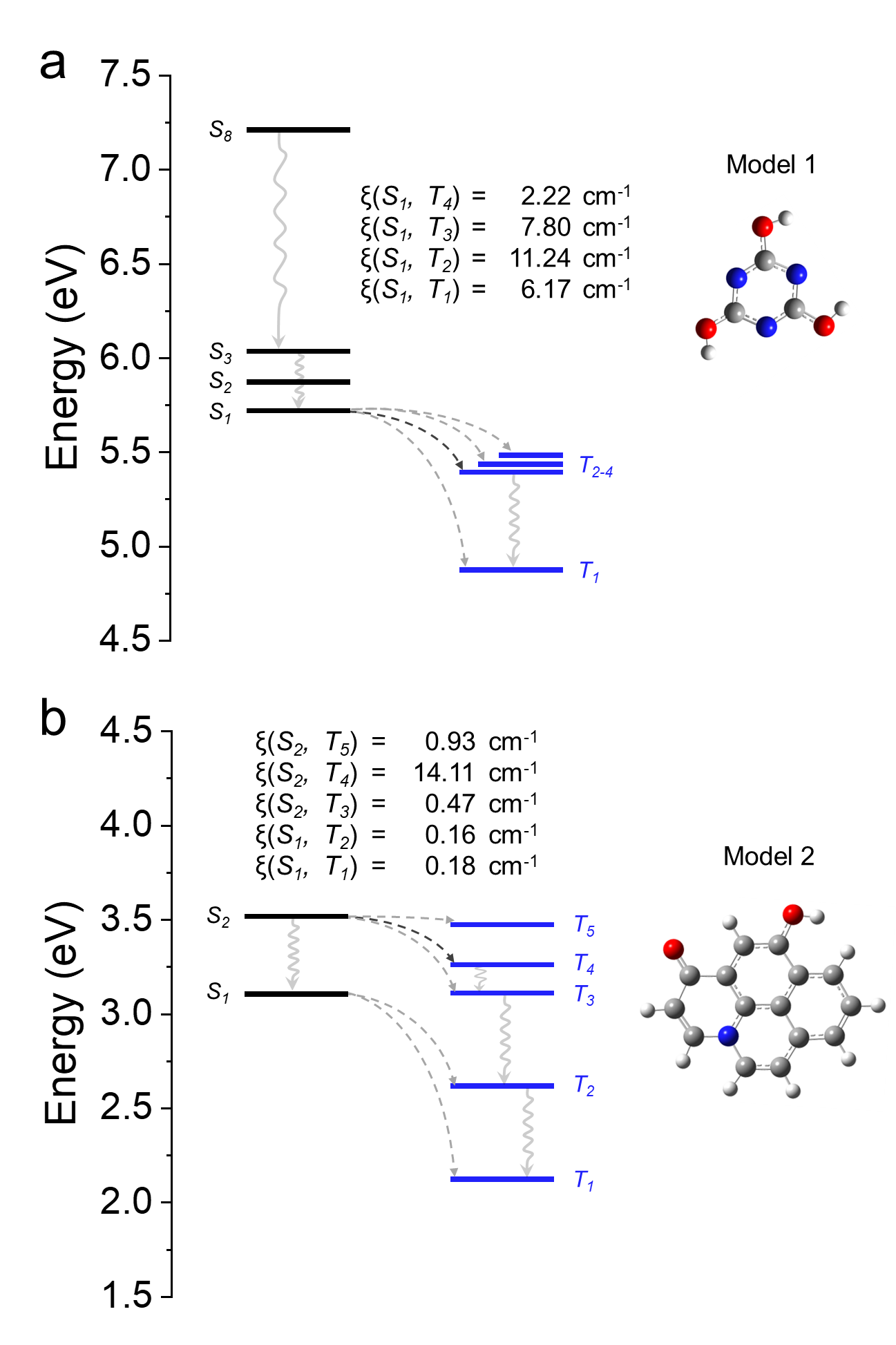
**

**Figure S11.** The calculated energy-level difference diagrams and spin-orbit coupling constants.

**Table S1.** XPS contents of CDs before dialysis.

|  |  | BU-CDs (%) | BUL-CDs (%) |
| --- | --- | --- | --- |
| B 1s | B-N | 3.56 | 2.97 |
|  | B-O | 2.34 | 2.06 |
| C 1s | C-C/C=C | 4.36 | 4.24 |
|  | C-N/C-O | 0.97 | 2.04 |
|  | C=O | 3.93 | 4.87 |
| N 1s | B-N | 2.63 | 3.81 |
|  | Pyridinic N | 2.44 | 3.79 |
|  | Pyrrolic N | 5.03 | 4.63 |
|  | Graphitic N | 3.76 | 3.32 |
| O 1s | C=O | 20.28 | 23.81 |
|  | B-O | 26.51 | 30.82 |
|  | C-O | 23.92 | 12.05 |
| F 1s | C-F | - | 1.59 |

**Table S2.** XPS contents of CDs after dialysis.

|  |  | BU-CDs (%) | | BUL-CDs (%) | |
| --- | --- | --- | --- | --- | --- |
| C 1s | C-C/C=C | 23.74 | 46.55 | 30.96 | 58.01 |
|  | C-N/C-O | 9.01 |  | 19.55 |  |
|  | C=O | 11.27 |  | 6.85 |  |
|  | π-π* | 1.99 |  | 0.65 |  |
| N 1s | Pyridinic N | 15.03 | 31.91 | 7.87 | 21.41 |
|  | Pyrrolic N | 11.21 |  | 7.43 |  |
|  | Graphitic N | 3.33 |  | 5.72 |  |
|  | π-π* | 2.34 |  | 0.39 |  |
| O 1s | C=O | 14.35 | 22.09 | 9.58 | 19.85 |
|  | C-O | 7.74 |  | 10.27 |  |
| F 1s | C-F | - | - | 0.73 | 0.73 |

**Table S3.** Comparison of the reported initial afterglow brightness of luminescent materials.

|  | Samples | Ex. Sources (nm) / Type | Ex. Power (Power density) | Afterglow peak (nm) | Initial afterglow brightness  (cd m^-2^) | Ref. |
| --- | --- | --- | --- | --- | --- | --- |
| CDs-based  materials | RhB-CDs@u/SiO_2_ | 365  UV flashlight | 5 W | 600 | 37.7 | ^[4]^ |
|  | Y-CDs@u/SiO_2_ |  |  | 575 | 24.21 |  |
|  | NIR-CDs@u/SiO_2_ |  |  | 680 | 4.97 |  |
|  | uCDs@SiO_2_@NaOH | 365  UV flashlight | 5 W | 540 | 39.53 | ^[5]^ |
|  | uCDs@SiO_2_ |  |  | 560 | 11.81 |  |
|  | uCDs | 365  UV flashlight | 5 W | 570 | 5.51 | ^[6]^ |
|  | uCDs@SiO_2_ |  |  | 570 | 50.93 |  |
| Rare earth-based materials | Sr_4_Al_14_O_25_:Eu^2+^, Dy^3+^ | 365  N/A* | N/A | 510 | 99.8 | ^[7]^ |
|  | CdSe/CdS:Eu^3+^@SrAl_2_O_4_ (98/2) | 362  N/A | N/A | 612 | 4.7 | ^[8]^ |
|  | CdSe/CdS:Eu^3+^@SrAl_2_O_4_ (85/15) |  |  | 522 | 5.73 |  |
|  | PC/LPP | White diode | 3.5 W | 516 | 4.9 | ^[9]^ |
|  | PA-NPs | 540  LED | 50  mW cm^-2^ | 615 | 10.7 | ^[10]^ |
| Organic-based materials | S-BINAP/gCLA | 300  N/A | N/A | 520 | 49.4 | ^[11]^ |
|  | R-BINAP/gCLA |  |  | 520 | 42.7 |  |
|  | S-BINAP/BN3/gCLA |  |  | 587 | 13.5 |  |
|  | R-BINAP/BN3/gCLA |  |  | 587 | 12.6 |  |
|  | TpPX@SEBS | 365  N/A | 65  mW cm^-2^ | 500 | 6 | ^[12]^ |
|  | PBHDB/PMMA | 280  N/A | N/A | 494 | 0.01 | ^[13]^ |
|  | DPhCzT | 254  N/A | N/A | 530 | 0.053 | ^[14]^ |
|  |  | 365  N/A |  | 530 | 0.061 |  |
|  |  | Sunlight  N/A |  | 530 | 0.134 |  |
|  | HCCP/HHTP/TA/PVA | 280  N/A | N/A | 482 | 0.223 | ^[15]^ |
|  | PMMA-11,12-PSICZ | 365  UV flashlight | 30 W | 444 | 2 | ^[16]^ |
|  | TN/TPBi | 365  LED | 5 mW cm^−2^ | 575 | 0.0136 | ^[17]^ |
|  | TpNP/PU | Mechano-responsive**^#^**  N/A | N/A | 504 | 0.611 | ^[18]^ |
| CDs-based  materials | BL-CDs | 365  UV flashlight | 61  mW cm^-2^ | 500 | 323 ± 3.0 | Our work |
|  | BUL-CDs |  |  | 531 | 406 ± 5.8 |  |

*N/A: Not applicable. **^#^**Mechano-responsive ultralong phosphorescence. Ex.: Excitation.

**Table S4.** Afterglow lifetime of CDs.

|  |  | τ_1_ (ms)/A_1_ (%) | τ_2_ (ms)/A_2_ (%) | τ_3_ (ms)/A_3_ (%) | τ_avg_ (ms) |
| --- | --- | --- | --- | --- | --- |
| BU-CDs | 470 nm | 5.609/9.29 | 50.00/41.08 | 200.0/49.63 | 120.32 |
| BUL-CDs | 520 nm | 53.20/11.66 | 239.3/43.03 | 703.6/45.31 | 427.98 |
|  | 540 nm | 35.35/7.87 | 202.3/39.72 | 577.9/52.41 | 386.01 |
|  | 650 nm | 86.05/22.77 | 267.3/54.97 | 800.0/19.27 | 320.69 |

**Table S5.** Energy level transition information of S_0_-S_n_ (n=1-10) in Model 1.

| Excited State | Energy Level (eV) | Oscillator Strength / f | Transition Configurations |
| --- | --- | --- | --- |
| S_1_ | 5.7136 | 0.00000 | H→L 96.7% |
| S_2_ | 5.8731 | 0.00410 | H→L+1 98.1% |
| S_3_ | 6.0323 | 0.00090 | H-1→L 52.3%, H-2→L+1 47.1% |
| S_4_ | 6.1285 | 0.00130 | H-3→L+1 77.1%, H-3→L 20.1% |
| S_5_ | 6.1650 | 0.00470 | H-3→L 78.8%, H-3→L+1 19.8% |
| S_6_ | 6.8799 | 0.00140 | H-2→L 53.6%, H-1→L+1 43.7% |
| S_7_ | 7.0841 | 0.20410 | H-1→L+1 29.0%, H-2→L 22.9%, H-2→L+1 20.3%, H-1→L 17.7% |
| S_8_ | 7.2070 | 0.25990 | H-2→L+1 27.1%, H-1→L 23.6%, H-1→L+1 20.2%, H-2→L 16.6%, H-4→L 7.5% |
| S_9_ | 7.8533 | 0.19500 | H-4→L 87.8% |
| S_10_ | 7.8991 | 0.16510 | H-4→L+1 89.7% |

**Table S6.** Energy level transition information of S_0_-S_n_ (n=1-10) in Model 2.

| Excited State | Energy Level (eV) | Oscillator Strength / f | Transition Configurations |
| --- | --- | --- | --- |
| S_1_ | 3.1021 | 0.10090 | H→L 77.9%; H→L+1 16.7% |
| S_2_ | 3.5231 | 0.19180 | H→L+1 69.1%; H→L 16.9%; H−3→L 5.0% |
| S_3_ | 3.6373 | 0.00110 | H−1→L 59.4%; H−1→L+1 34.6% |
| S_4_ | 3.9616 | 0.05050 | H→L+2 89.0%; H→L+1 5.4% |
| S_5_ | 4.0816 | 0.00020 | H−1→L+1 59.8%; H−1→L 36.9% |
| S_6_ | 4.4530 | 0.01110 | H−1→L+3 65.9%; H−2→L 22.8%; H−3→L 8.3% |
| S_7_ | 4.6246 | 0.05150 | H−2→L 69.4%; H−1→L+3 18.6%; H−4→L 5.6% |
| S_8_ | 4.8953 | 0.09390 | H−3→L 68.3%; H−4→L+1 10.5%; H→L+3 7.9% |
| S_9_ | 4.9303 | 0.00000 | H−1→L+2 97.1% |
| S_10_ | 4.9600 | 0.00470 | H−2→L+1 82.0%; H−4→L 10.5% |

**Reference**

[1] Y. Liu, D. Cheng, B. Wang, J. Yang, Y. Hao, J. Tan, Q. Li, S. Qu, Carbon Dots-Inked Paper with Single/Two-Photon Excited Dual-Mode Thermochromic Afterglow for Advanced Dynamic Information Encryption, *Adv. Mater.* **2024**, *36*, 2403775.

[2] T. Lu, A comprehensive electron wavefunction analysis toolbox for chemists, Multiwfn, *J. Chem. Phys.* **2024**, *161*.

[3] T. Lu, F. Chen, Multiwfn: A multifunctional wavefunction analyzer, *J. Comput. Chem.* **2012**, *33*, 580–592.

[4] J. Zhu, C. Li, Y. Zhu, J. Hu, Y. Nan, X. Chen, K.-K. Liu, H. Wang, C. Shan, W. Xu, Q. Lou, Long-Wavelength Afterglow Emission with Nearly 100% Efficiency through Space-Confined Energy Transfer in Organic-Carbon Dot Hybrid, *Nano Lett.* **2024**, *24*, 13307–13314.

[5] Y. Nan, Z. Li, C. Li, Y. Zhu, X. Ma, Z. Zhu, J. Hu, Y. Zhu, G. Zheng, J. Zhu, Construction of Multicolor Phosphorescent Carbon Dots via Multiple Confinement Strategies, *Inorg. Chem.* **2025**, *64*, 7499–7507.

[6] Y. Zhu, C. Li, J. Zang, G. Zheng, Y. Nan, X. Xia, Z. Zhu, G. Cao, J. Hu, J. Zhu, Enhanced afterglow performance of carbon dots via surface modification engineering, *J. Lumin.* **2025**, *283*, 121271.

[7] J. Du, J. Zhang, T. Wang, P. Zhou, L. Cao, Q. Liu, H. Lin, Brighten strontium aluminate long-persistence materials via optimizing defect energy level distribution, *Mater. Today Phys.* **2023**, *38*, 101229.

[8] Z. Chen, Y. Li, X. Yuan, L. Wu, CdSe/CdS:Eu3+@SrAl2O4:Eu2+, Dy3+ phosphor composite shows red emitting luminescence with high brightness and long lifetime based on efficient PRET and color superposition, *Inorg. Chem. Commun.* **2025**, *173*, 113882.

[9] Y. Ding, Q. Wu, Z. Wang, S. Yin, B. Zhang, J. Yang, Tunable multicolor photonic crystal/long persistent phosphor composites for dual-mode display in safety sign and information encryption, *Mater. Today Chem.* **2025**, *45*, 102632.

[10] M. Xu, J. Liu, X. Su, Q. Zhou, H. Yuan, Y. Wen, Y. Cheng, F. Li, Lanthanide-containing persistent luminescence materials with superbright red afterglow and excellent solution processability, *Sci. China Chem.* **2021**, *64*, 2125–2133.

[11] J. Zhang, S. Zhang, C. Sun, R. Wang, Z. Guo, D. Cui, G. Tang, D. Li, J. Yuan, X. Lu, C. Zheng, W. Huang, R. Chen, Highly Bright Pure Room Temperature Phosphorescence for Circularly Polarized Organic Hyperafterglow, *Adv. Mater.* **2025**, *37*, 2500953.

[12] T. Tang, D. Guo, J. Chen, K. Zhang, F. Lin, H. Dai, D. Liu, Y. Zhang, Z. Chi, Z. Yang, H. Huang, Robust Ultra-high Phosphorescence Brightness from Phenyl(triphenylen-2-yl)methanone Derivatives Doped Non-polar Polymers, *Chin. J. Chem.* **2025**, *n/a*, DOI: 10.1002/cjoc.70237.

[13] C. Wang, L. Qu, X. Chen, Q. Zhou, Y. Yang, Y. Zheng, X. Zheng, L. Gao, J. Hao, L. Zhu, B. Pi, C. Yang, Poly(arylene piperidine) Quaternary Ammonium Salts Promoting Stable Long-Lived Room-Temperature Phosphorescence in Aqueous Environment, *Adv. Mater.* **2022**, *34*, 2204415.

[14] Z. An, C. Zheng, Y. Tao, R. Chen, H. Shi, T. Chen, Z. Wang, H. Li, R. Deng, X. Liu, W. Huang, Stabilizing triplet excited states for ultralong organic phosphorescence, *Nat. Mater.* **2015**, *14*, 685–690.

[15] Y. Zhang, X. Chen, J. Xu, Q. Zhang, L. Gao, Z. Wang, L. Qu, K. Wang, Y. Li, Z. Cai, Y. Zhao, C. Yang, Cross-Linked Polyphosphazene Nanospheres Boosting Long-Lived Organic Room-Temperature Phosphorescence, *J. Am. Chem. Soc.* **2022**, *144*, 6107–6117.

[16] C. Li, F. Guo, Y. Zhu, Q. Zhou, Q. Chen, Y. Wang, J. Huang, L. Qu, C. Yang, Ink-Free Screen Printing in Water Environment-Based Slightly Cross-Linked Polymer Phosphorescence Systems, *Macromolecules* **2023**, *56*, 10028–10036.

[17] C. Lin, Z. Wu, H. Ma, J. Liu, S. You, A. Lv, W. Ye, J. Xu, H. Shi, B. Zha, W. Huang, Z. An, Y. Zhuang, R.-J. Xie, Charge trapping for controllable persistent luminescence in organics, *Nat. Photonics* **2024**, *18*, 350–356.

[18] J. Chen, F. Lin, D. Guo, T. Tang, Y. Miao, Y. Wu, W. Zhai, H. Huang, Z. Chi, Y. Chen, Z. Yang, In Situ Reversible and Robust Mechano-Responsive Ultralong Phosphorescence of Polyurethane Elastomer, *Adv. Mater.* **2024**, *36*, 2409642.
